# Integrated Multi-Omics Convergence Scoring Reveals Modular Network Architecture with Age-Associated Mitochondrial Signatures in Sarcopenia

**DOI:** 10.3390/ijms27219500

**Published:** 2026-10-24

**Authors:** Dahyun Park, Jiwon Park, Ock K. Chun, Eun-Young Kwon, Heekyong R. Bae

**Affiliations:** 1 Center for Food and Nutritional Genomics, Kyungpook National University, Daegu 41566, Republic of Korea; 2 Department of Food Science and Nutrition, Kyungpook National University, Daegu 41566, Republic of Korea; 3 Department of Nutritional Sciences, University of Connecticut, 3624 Horsebarn Road Extension Unit 4017, Storrs, CT 06269-4017, USA; ock.chun@uconn.edu; 4 Center for Beautiful Aging, Kyungpook National University, Daegu 41566, Republic of Korea

**Keywords:** sarcopenia, multi-omics integration, skeletal muscle transcriptome, mitochondria, ageing, DNA methylation, genome-wide association study, network analysis, replication

## Abstract

Sarcopenia arises from convergent genetic, epigenetic, transcriptomic, metabolic, and microbiome-associated perturbations, yet conventional multi-omics analyses often yield fragmented, layer-specific feature lists. We developed an integrated multi-omics convergence scoring framework that calculates an Integrated Convergence Score (ICS) by combining standardised layer-level statistical evidence, layer-specific pathway context, network features and sarcopenia-specific disease-relevance evidence. We applied it to skeletal muscle transcriptomes from five independent cohorts (208 participants), ageing-associated muscle DNA methylation, and genome-wide association studies of muscle mass and strength, each analysed against its own null distribution. Gene-level changes were few and did not replicate, whereas mitochondrial respiration, mitochondrial translation and contraction pathway scores were lower in sarcopenia in the pooled analysis. These mitochondrial signatures were also lower in ageing-, frailty-, and pre-sarcopenia-related comparisons, indicating a component shared with muscle ageing. The three layers implicated largely distinct mechanisms. Compared with rank-based combined statistics, the ICS top-ranked genes formed a more connected network, which partly reflects the network component of the score. A context map placed the modules within mitochondrial respiration, creatine-based energy buffering, and NAD–PGC-1α signalling. This framework organises fragmented multi-omics evidence into a systems-level gene network associated with sarcopenia and prioritises candidate genes and mechanisms for future functional studies.

## 1. Introduction

Sarcopenia extends beyond a simple age-associated decline in skeletal muscle mass. It is defined by low muscle strength and mass with impaired physical performance, is associated with falls, disability, and mortality, and is increasingly recognised as a complex systems-level disorder in which genetic susceptibility, epigenetic remodelling, cellular senescence, metabolic imbalance, mitochondrial dysfunction, and gut-associated perturbations converge to impair muscle strength and physical function [1,2]. Recent high-resolution studies have reinforced this multilayered view of muscle ageing. Single-nucleus and multimodal atlases of ageing human skeletal muscle have revealed cell-type-specific senescence programmes and niche remodelling [3,4,5]. Integrative genomic, epigenomic, and telomere-length analyses have connected sarcopenia-related biology to frailty [6] and to genetic mechanisms shared with other age-related diseases [7]. In parallel, current mechanistic frameworks describe muscle ageing as the convergence of senescence, inflammaging, mitochondrial and proteostatic failure, impaired regeneration, and tissue-niche remodelling [8]. Among these processes, reduced mitochondrial oxidative capacity and NAD^+^ biosynthesis have been reported in sarcopenic muscle across ethnicities [9], but whether such mitochondrial changes are specific to sarcopenia or part of muscle ageing more broadly remains unclear.

Despite rapid advances in high-throughput omics technologies, translating multi-omics data into biologically interpretable sarcopenia mechanisms remains challenging. Genetic variants identified through genome-wide association studies (GWAS) are often difficult to connect to downstream changes in RNA expression, protein activity, metabolite abundance, or tissue-level dysfunction [10]. Previous multi-omics studies have also remained largely descriptive, generating lists of differentially expressed genes, altered proteins, metabolites, or microbial taxa without clearly distinguishing coherent disease modules from secondary markers of tissue injury [10,11,12]. Two further problems arise with public sarcopenia data. Muscle transcriptome cohorts are small and differ in diagnostic criteria, ethnicity, sex, age, and platform, so gene-level findings from one cohort often fail to replicate in another. In addition, the layers measure different biology: muscle transcriptomes describe the current state of the tissue, methylation datasets often compare older with younger muscle and therefore record an ageing imprint, and GWAS of muscle mass and strength reflect inherited predisposition. Treating such layers as interchangeable evidence for the same genes risks double counting and spurious convergence. These limitations are particularly relevant to sarcopenia, whose development and progression likely reflect the combined effects of multiple interacting biological processes rather than a single dominant molecular defect.

Recent analytical developments have begun to address this gap. Integrative multi-omics analyses have nominated key targets and cellular mechanisms for intervention in sarcopenia [13], explainable graph neural network models have shown that prior biological knowledge and network topology can improve multi-omics biomarker discovery [14], and attention-aware multi-task learning has nominated candidate targets for drug repurposing in sarcopenia [15]. Such learning-based methods, however, may depend on training labels that do not fully match the biology captured by the data and are not always compared with simple baselines. At the other end, statistical combination of layer evidence by weighted Z-score (Stouffer) is transparent and well characterised [16], but it treats each gene independently and ignores the pathways and interactions through which layers may connect. Moreover, when curated disease genes or mechanism priors are added to rankings or networks, recovery of known biology becomes partly circular. A remaining need is therefore to integrate statistical evidence, pathway context, network topology, and disease-specific biological context within a transparent framework that generates not only ranked candidate genes but also a mechanistically organised gene-network map, and that is tested directly against conventional combined statistics.

To address this need, we developed an integrated multi-omics convergence scoring approach for sarcopenia target prioritisation and gene-network mapping. Using five independent skeletal muscle transcriptome cohorts with patient-level data, we first asked whether sarcopenia is reflected more consistently at the level of genes or of mechanisms, and how mechanism-level changes relate to ageing, frailty and pre-sarcopenia. We then analysed the transcriptome, ageing-associated methylation, and GWAS layers separately, each against a null distribution that preserves its own dependence structure. Rather than relying on a single statistical threshold or predictive model, we calculated an Integrated Convergence Score (ICS) that combines, with equal weights, cross-layer statistical evidence, a sarcopenia-specific clinical-trait component, layer-specific mechanism evidence, and network connectivity. We compared the ICS with rank-based Stouffer combination of the same layer scores for network structure across progressively expanded top-ranked gene sets and connectivity between genes supported by different layers. Finally, we placed the resulting modules in context with faecal and blood metabolome themes and literature-based gut microbe associations, without using these data to rank genes. We hypothesised that integrating layer-specific mechanisms and network information would yield a more connected set of top-ranked genes, linking genes supported by different layers, than combining gene-level statistics alone.

## 2. Results

### 2.1. Study Design: Layer-Specific Analysis Followed by Integration and Direct Comparison

**Figure 1:**
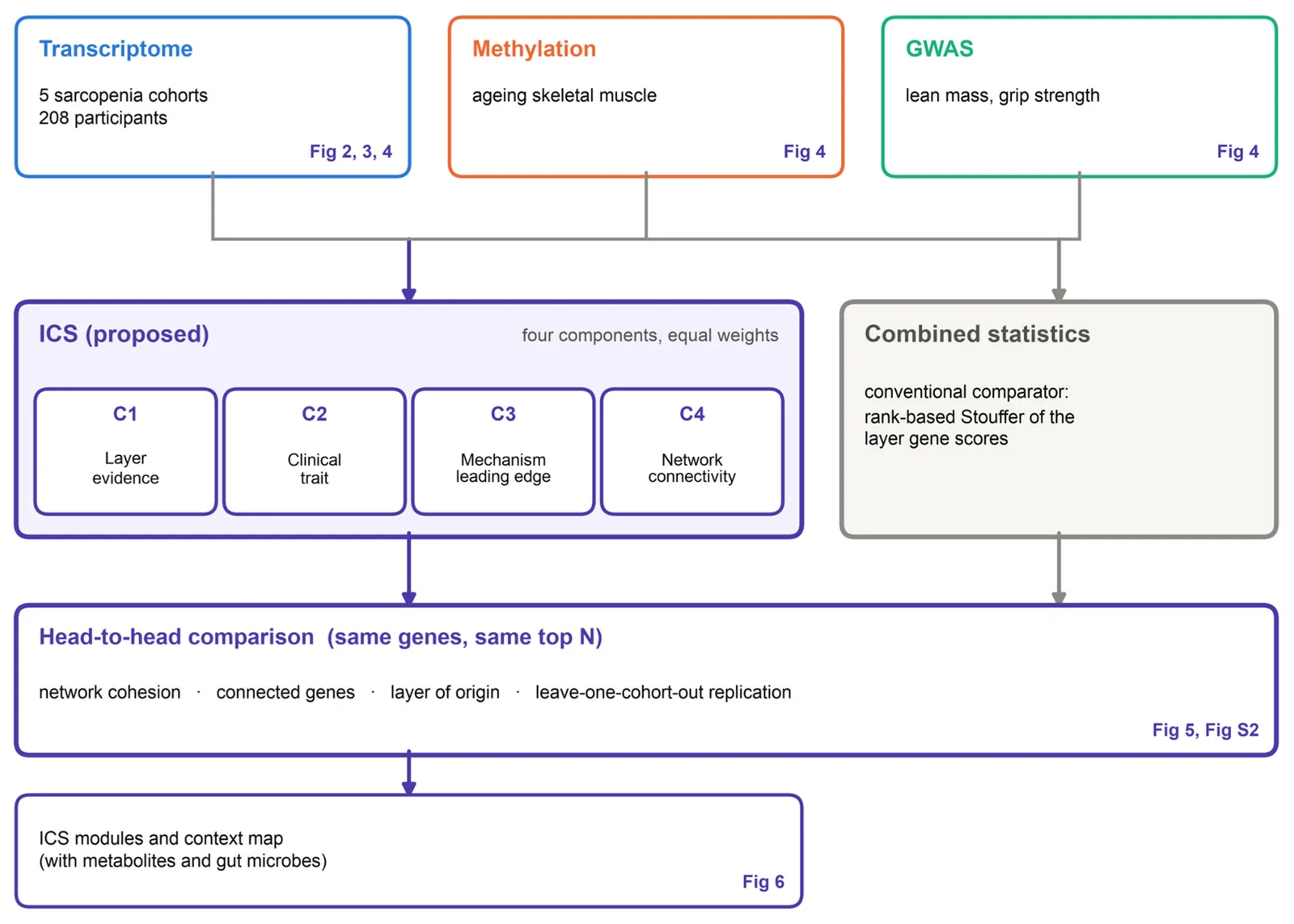
Study design. Three omics layers (transcriptome of 208 participants in five cohorts, ageing methylation, GWAS) were analysed separately, each against its own null distribution. The same layer results were then integrated in two ways: by the integrated convergence score (ICS; four equally weighted components, C1–C4) and by conventional combined statistics (rank-based Stouffer). The two rankings were compared over the same genes and top N. The ICS result was placed in context with metabolite and gut microbe findings. Purple tags indicate the figure showing each result. Box colours indicate the transcriptome (blue), methylation (orange) and GWAS (green) layers; purple and grey indicate the ICS and combined-statistics analyses, respectively.

The study design is summarised in Figure 1. Three primary omics layers from public human data were analysed separately, and the same layer results were then integrated in two ways. The first was the integrated convergence score (ICS), with four equally weighted components: layer evidence (C1), clinical trait (C2), mechanism leading edge (C3), and network connectivity (C4). The second was conventional combined statistics, that is, rank-based Stouffer combination of the layer gene scores.

The first primary layer comprised skeletal muscle transcriptomes from five independent sarcopenia cohorts in China, Singapore, the United Kingdom, Jamaica, and Canada. The main analyses included 208 participants (111 healthy, 77 sarcopenia, and 20 pre-sarcopenia); 24 participants meeting a single criterion were analysed separately (Figure S1). The second layer was ageing-associated skeletal muscle DNA methylation. The third layer was GWAS of appendicular lean mass and low grip strength.

Both rankings used the same underlying omics layers, while the ICS additionally incorporated clinical-trait, mechanism, and network information. The two rankings were compared over the same genes and the same top N for network structure and layer of origin. Faecal and blood metabolome data and literature-based gut microbe associations were not used in the ranking. They were used afterwards to place the ICS result in context.

### 2.2. Five Independent Muscle Transcriptome Cohorts Show Lower Mitochondrial Pathway Scores in Sarcopenia

#### 2.2.1. Few Gene-Level Changes but Consistent Mechanism-Level Changes

**Figure 2:**
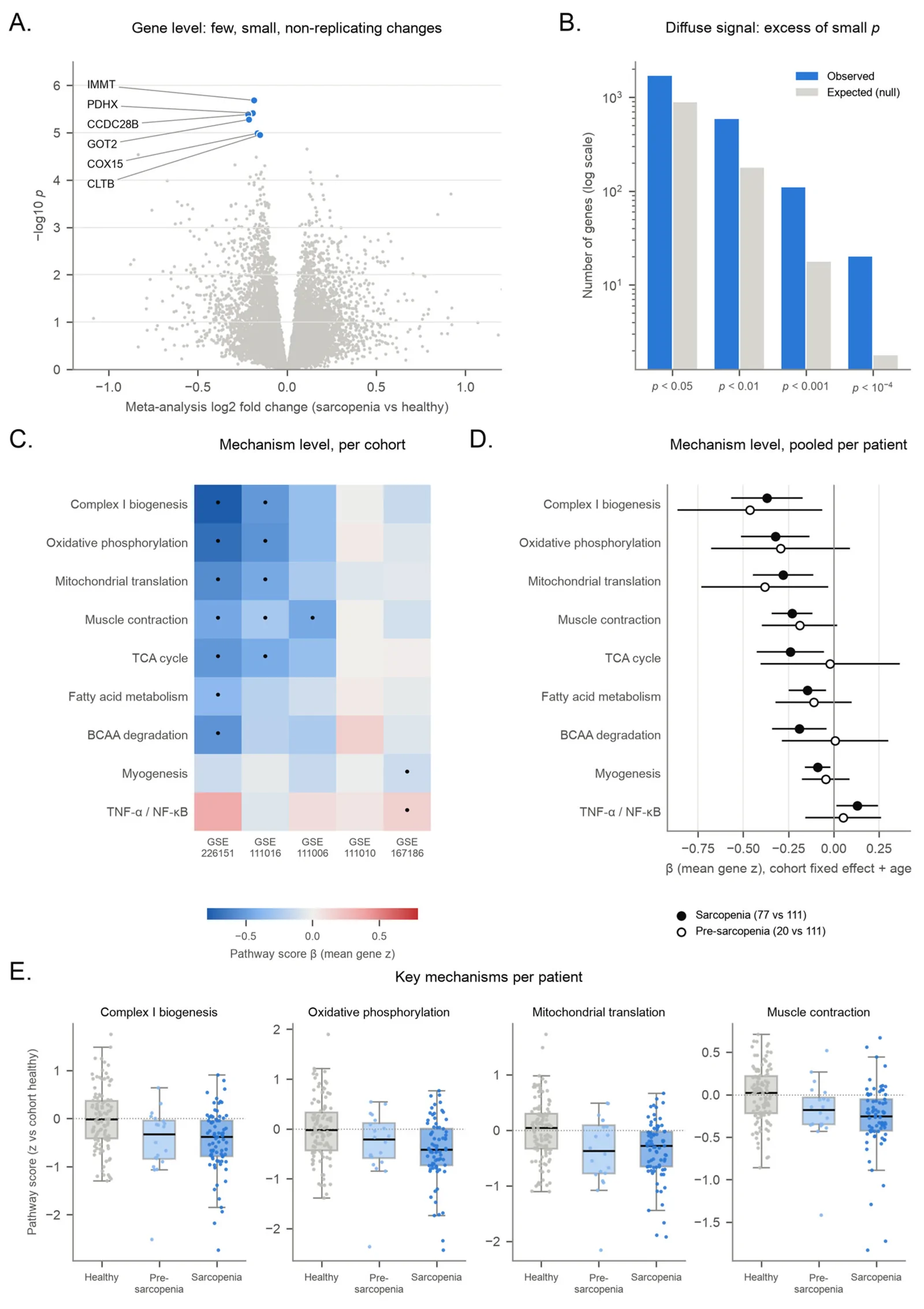
Sarcopenic muscle shows few gene-level changes but consistent mechanism-level changes. (**A**) Gene-level random-effects meta-analysis of five cohorts (sarcopenia vs. healthy; 17,712 genes); genes at FDR < 0.05 are labelled and shown in blue. (**B**) Number of genes below each *p* threshold, observed (blue) vs. expected under the null (grey). (**C**) Pathway scores per cohort (sarcopenia vs. healthy; colour scale, β in units of mean gene z); dots, *p* < 0.05. (**D**) Pooled participant-level estimates (β ± 95% CI, mean gene z) for sarcopenia (filled; 77 vs. 111 healthy) and pre-sarcopenia (open; 20 vs. 111). (**E**) Scores of four key mechanisms per patient; boxes show the median and interquartile range, whiskers extend to the most extreme values within 1.5 × the interquartile range of the box, and the dotted line marks the healthy mean (grey, healthy; light blue, pre-sarcopenia; blue, sarcopenia).

Few genes reached statistical significance in the gene-level meta-analysis. In the random-effects meta-analysis of the five cohorts (17,712 genes), six genes reached FDR < 0.05 (*IMMT*, *PDHX*, *CCDC28B*, *GOT2*, *COX15*, and *CLTB*); their median absolute log2 fold change was 0.19, compared with 0.06 across all genes (Figure 2A). After the Hartung–Knapp–Sidik–Jonkman adjustment, all six genes remained nominally significant (*p* = 0.0004–0.009), but none met the FDR < 0.05 threshold. Genes significant in the largest cohort (GSE226151) showed limited directional agreement in the other cohorts (43–61% changed in the same direction in each cohort).

Nevertheless, more genes than expected under the null had small *p* values (*p* < 0.05: 1696 vs. 886 expected; *p* < 0.001: 109 vs. 18; *p* < 10^−4^: 20 vs. 1.8). This excess was consistent with a distributed transcriptomic signal (Figure 2B).

At the mechanism level, the changes were largely consistent across cohorts. Patient-level pathway scores (mean gene z relative to each cohort’s healthy participants) were lower in sarcopenia for mitochondrial pathways and muscle contraction in most cohorts (Figure 2C). In the pooled patient-level model with cohort fixed effect and age, complex I biogenesis (β = −0.37, *p* = 2.5 × 10^−4^), oxidative phosphorylation, mitochondrial translation, the TCA cycle, and muscle contraction (β = −0.23, *p* = 4.4 × 10^−5^) were all lower in sarcopenia (Figure 2D; β in units of mean gene z). Complex I and mitochondrial translation were already lower at the pre-sarcopenia stage. Patient-level distributions for the four key mechanisms are shown in Figure 2E.

Two sensitivity analyses addressed control definition and sex. The first concerned the control definition in GSE111010. In the sarcopenia status deposited with the dataset, the control group included participants meeting one sarcopenia criterion. With that control group, the direction of oxidative phosphorylation in this cohort reversed (mean z +0.29 vs. −0.08 with controls meeting no criterion). The overall direction of the five-cohort result was retained (meta z −1.41 with 5/5 cohorts concordant using controls meeting no criterion vs. −1.27 with 4/5 using the deposited status).

The second sensitivity analysis was restricted to men. Excluding the women of GSE226151, the mitochondrial and myogenesis results remained similar (oxidative phosphorylation, meta-analysis of DESeq2-based pathway z: −1.45 vs. −1.55 for all participants).

#### 2.2.2. Mitochondrial Signals Are Lower Across Ageing, Frailty and Sarcopenia Comparisons

**Figure 3:**
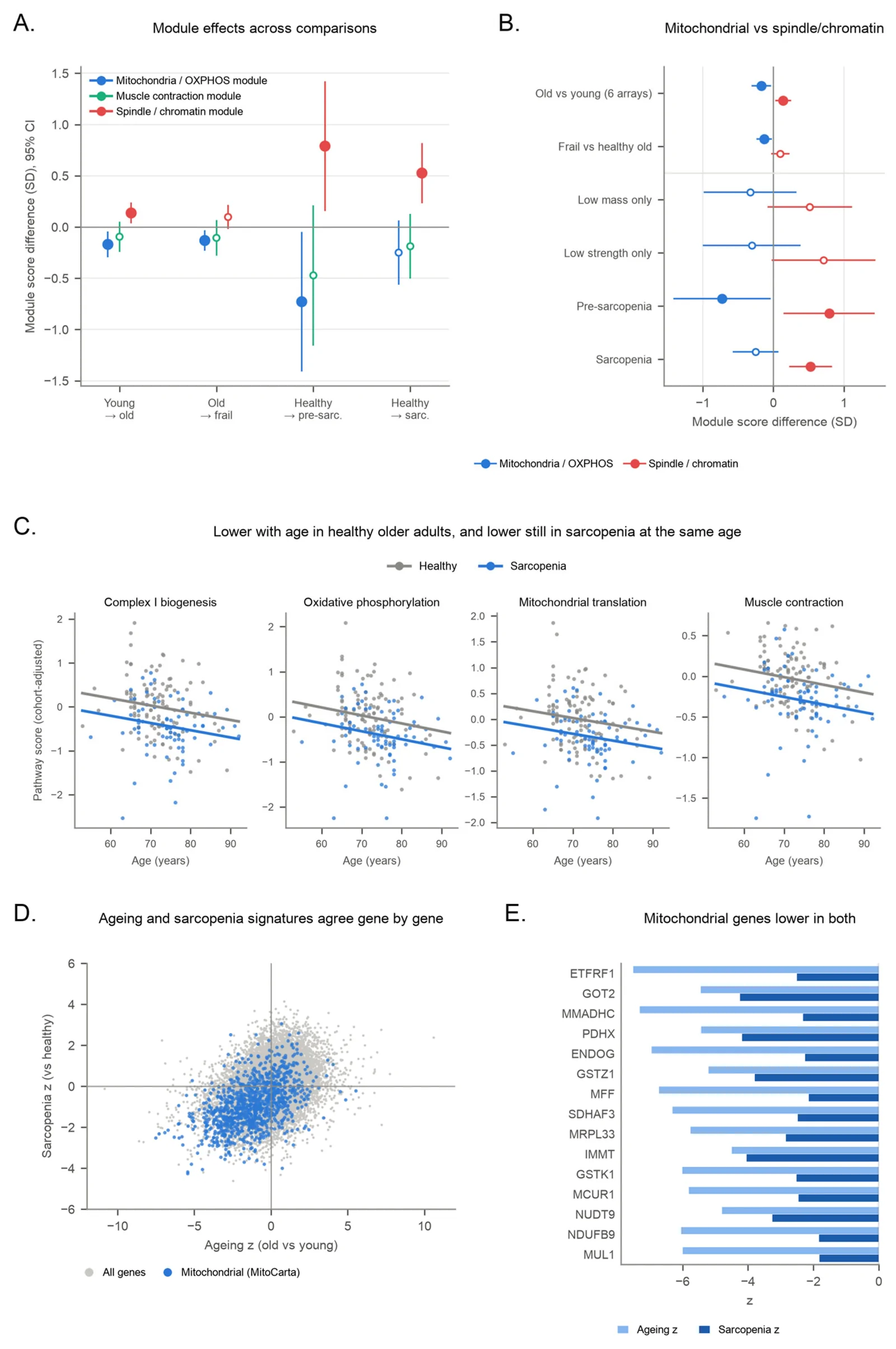
Mitochondrial signals are lower across ageing-, frailty- and sarcopenia-related comparisons. (**A**) Co-expression module scores in four separate comparisons (old vs. young, frail vs. healthy older, pre-sarcopenia vs. healthy, sarcopenia vs. healthy); estimate ± 95% CI in module SD units; filled points, *p* < 0.05; open points, *p* ≥ 0.05. The comparisons involve different groups and are not a time series. (**B**) Mitochondria/OXPHOS and spindle/chromatin modules across all contrasts, including single-criterion groups. (**C**) Pathway scores (mean gene z) against age (healthy, n = 111; sarcopenia, n = 77), adjusted for cohort, with OLS fits. (**D**) Gene-level agreement between the ageing and sarcopenia signatures; mitochondrial genes (MitoCarta 3.0) in blue. (**E**) The 15 mitochondrial genes most decreased in both signatures.

To ask whether the mitochondrial signal is specific to sarcopenia or shared with muscle ageing, consensus co-expression modules were related to ageing, frailty, pre-sarcopenia and sarcopenia (Figure 3A,B). The mitochondria/OXPHOS module (1740 genes) was lower in all four comparisons: with ageing (−0.17 SD, *p* = 0.009, FDR = 0.064), with frailty (−0.13 SD, *p* = 0.009, FDR = 0.063), at pre-sarcopenia (−0.73 SD, *p* = 0.036, FDR = 0.067) and in sarcopenia (−0.25 SD, *p* = 0.12, FDR = 0.33). These comparisons involve different groups and control groups, so they show a shared direction rather than a stepwise progression; the pre-sarcopenia estimate, from a single small cohort, was larger than the sarcopenia estimate.

In contrast, the spindle/chromatin module was higher at pre-sarcopenia (+0.79 SD, *p* = 0.015) and in sarcopenia (+0.53 SD, *p* = 4.3 × 10^−4^, FDR = 0.009), with a smaller ageing effect (+0.14 SD) and no statistically significant association with frailty; whether these effects differ from one another was not formally tested. Participants meeting only the low-mass or only the low-strength criterion showed the same directions without reaching significance (Figure 3B).

Within this study’s participants, pathway scores were also related to chronological age (Figure 3C; β in units of mean gene z; models with cohort, without sex). In healthy older adults, mitochondrial and contraction pathway scores were lower with age (per 10 years: complex I −0.22, *p* = 0.046; oxidative phosphorylation −0.23, *p* = 0.029; muscle contraction −0.14, *p* = 0.012).

At the same age, sarcopenia was associated with further reductions in these scores. In this model, with age in years, the estimates were complex I −0.40 (*p* = 1.0 × 10^−4^), oxidative phosphorylation −0.35 (*p* = 3.5 × 10^−4^), and mitochondrial translation −0.31 (*p* = 3.6 × 10^−4^). These estimates differ slightly from Figure 2D, which uses within-cohort standardised age.

At the gene level, the ageing and sarcopenia signatures were moderately correlated. Spearman’s ρ was 0.36 across 14,315 genes and 0.50 across 1016 mitochondrial genes, 63% of which were lower in both signatures (Figure 3D). The mitochondrial genes most consistently decreased in both included *GOT2*, *PDHX*, and *IMMT*, which were also FDR-significant in the sarcopenia meta-analysis, together with *ETFRF1*, *MMADHC*, *ENDOG*, *MFF*, and *SDHAF3* (Figure 3E).

These cross-sectional data show lower mitochondrial transcript signals in ageing-, frailty-, and sarcopenia-related comparisons, consistent with a shared, ageing-related component. They do not establish a stepwise progression or that ageing causes the decline. The spindle/chromatin increase was most evident in the disease comparisons.

### 2.3. Different Omics Layers Mainly Reflect Different Mechanisms

**Figure 4:**
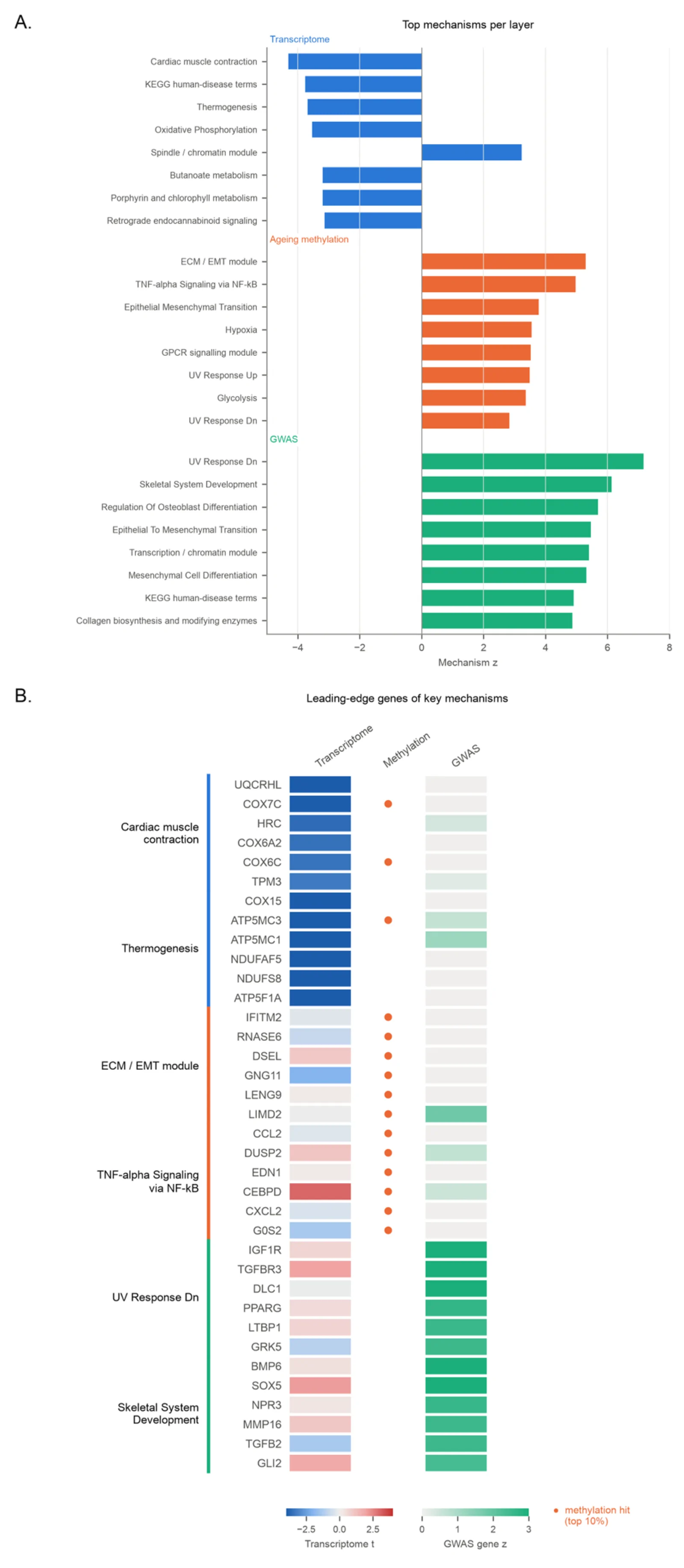
The three omics layers implicate largely distinct mechanisms. (**A**) Top eight mechanisms per layer at FDR < 0.05, ranked by |z| (bars coloured by layer); KEGG human-disease terms are collapsed into one row. For the transcriptome, negative z indicates lower values in sarcopenia. (**B**) Leading-edge genes of the top two named mechanisms per layer: transcriptome t (colour scale), methylation layer hit (dot; top 10% of the CpG-adjusted score), and GWAS gene z (colour scale). Gene lists are given in Table S1.

Each layer was tested against its own null distribution, and the three layers implicated largely distinct mechanisms (Figure 4A). The transcriptome, which reflects muscle state, yielded 18 mechanisms at FDR < 0.05. These centred on mitochondrial energy metabolism (oxidative phosphorylation, thermogenesis) and contraction, all lower in sarcopenia, and on the spindle/chromatin module, higher in sarcopenia.

Ageing methylation, which reflects the ageing imprint, yielded 13 mechanisms. These centred on inflammation, extracellular matrix, and hypoxia, including the ECM/EMT and GPCR signalling modules, TNF-α signalling via NF-κB, and epithelial–mesenchymal transition.

GWAS, which reflects predisposition, yielded 170 mechanisms. These centred on skeletal system development, osteoblast differentiation, and collagen biosynthesis, consistent with body-size biology.

Convergence of the layers on the same genes beyond expectation was not detected, whereas mechanism-level overlap exceeded expectation, mainly between the methylation and GWAS layers. Mechanisms nominally significant (*p* < 0.05) in two or more layers were more frequent than expected (266 vs. 205.4 expected; permutation *p* < 0.001), largely because of overlap between the ageing methylation and GWAS layers (153 vs. 82.5 expected), whereas the transcriptome overlapped with the other layers at close to chance level. The numbers of genes in the top 10% of two layers (361 vs. 340 expected) or all three layers (10 vs. 10.2) were close to expectation. The leading-edge genes driving each layer’s mechanisms were strong in that layer and weak in the others (Figure 4B; gene lists in Table S1). For example, respiratory-chain and contraction genes drove the transcriptome mechanisms; inflammatory and matrix genes, the methylation mechanisms; and growth-factor and developmental genes, the GWAS mechanisms.

The GWAS layer partly reflects body size. Nine of the 21 low grip strength loci colocalised with height or body mass index (PP4 > 0.8). Muscle eQTL colocalisation was found for a few genes only: *L3MBTL3* and *KCNH2* in GTEx, and *ZBTB38*, *MAP3K3*, *LRRC37A3* and one unannotated transcript at chromosome 16 in the FUSION study.

Thus, the main enrichment patterns differed between layers: genes rarely overlapped beyond chance, whereas mechanism-level overlap exceeded expectation, mainly between the methylation and GWAS layers. We interpret the layers as capturing different aspects of the disease (muscle state, ageing imprint, and predisposition), although differences in sample size, measured material, and power limit this inference. This motivated integration at the level of mechanisms and networks rather than by combining gene-level statistics.

### 2.4. ICS Multi-Omics Integration Identifies Top-Ranked Genes Connected Across Different Layers

#### 2.4.1. The ICS Top Genes Form a Connected Network Linking Layers

The ten top-ranked ICS genes were *MRPL34*, *PPIF*, *UQCC1*, *TPM2*, *EGR1*, *PROCR*, *SUMO1*, *TMEM176B*, *HINT1*, and *CDKN2C* (the full ranking is given in Table S2). The top 100 included 44 mitochondrial genes (MitoCarta 3.0, accessed on 1 October 2026) and 9 clinical-trait GWAS genes, 8 of which were tagged as sharing a body-size signal.

**Figure 5:**
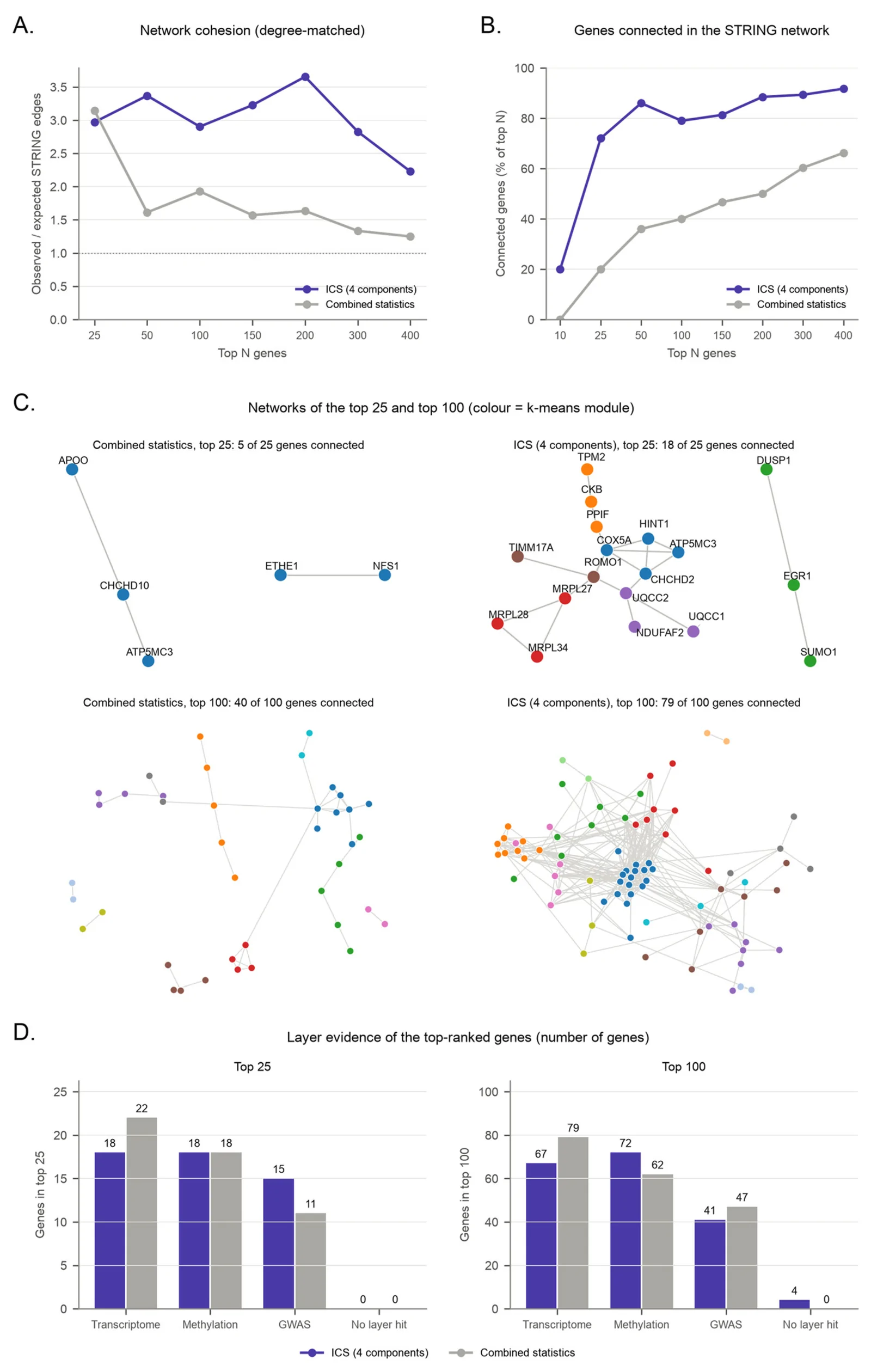
ICS compared with combined statistics. (**A**) Network cohesion of the top 25–400 genes (observed/expected STRING interactions against degree-matched random sets; purple, ICS; grey, combined statistics; dotted line, 1). Because the ICS includes a network component (C4), part of its higher connectivity is built in. (**B**) Percentage of top-N genes connected to another top-N gene. (**C**) STRING networks of the top 25 (gene names shown) and top 100 (colour, module); unconnected genes omitted. (**D**) Number of top-25 and top-100 genes in the top 10% of each layer, and with no layer hit.

Compared with combined statistics, the ICS top genes formed a more connected network at every list size and a more cohesive one from the top 50 onwards (Figure 5A,B). Network cohesion (observed/expected STRING interactions against degree-matched random gene sets) was 2.90 vs. 1.93 for the top 100 and 2.23 vs. 1.25 for the top 400. In terms of connected genes, 72% vs. 20% of the top 25 and 79% vs. 40% of the top 100 had at least one interaction (combined score ≥ 0.4) with another top gene.

In the networks of the top 25 and top 100, the ICS genes formed one main connected structure. It contained a respiratory chain–mitochondrial translation core linked to creatine kinase (*CKB*) and contraction (*TPM2*), with a small separate component of *SUMO1*, *EGR1*, and *DUSP1* (Figure 5C). The combined-statistics top 25, by contrast, contained only five connected genes (18 vs. 5 of 25; 79 vs. 40 of 100).

The ICS top genes also connected genes supported by different layers more often (post hoc analysis; Table S2, sheet i). Cross-layer interactions were defined as interactions in which each gene carries a layer hit that the other lacks. They numbered 11 vs. 1 among the top 25 and 113 vs. 16 among the top 100. Genes taking part in such interactions numbered 61 vs. 26 in the top 100. Examples found only among the ICS top genes include *PPIF*–*CKB*, *PPIF*–*PPARGC1A*, *UQCC1*–*UQCC2*, and *HINT1*–*COX5A*.

The layer origin of the top genes showed a similar pattern (Figure 5D). In the top 25, the ICS included more GWAS-supported genes (15 vs. 11), and 23 of the 25 genes had hits in two or more layers (vs 21). In the top 100, it included more methylation-supported genes (72 vs. 62), and four genes were ranked through the mechanism and network components alone, without any layer hit. The methylation count partly reflects the CpG-residual score, which favours genes with few CpGs.

Part of this network advantage is built into the score, because the ICS contains a network component. Removing the network component (C4) lowered top-100 cohesion to 0.73 times the degree-matched random expectation, and lowered connectivity at every size (Figure S3). The proportion of cross-layer interactions was similar between the rankings (34% vs. 39% in the top 100); the larger number came from the larger number of interactions overall.

An input permutation analysis used the fast version of the ICS (Supplementary Methods), whose top 100 differs slightly from the main ranking. When the layer values were shuffled across genes, the network structure of its top 100 did not differ significantly from the permutation distribution: cohesion 3.37 vs. a mean of 2.06 (*p* = 0.062); connected genes (experiments/databases interactions) 54 vs. 38 (*p* = 0.051) (Figure S4). The higher connectivity of the ICS top genes is therefore best regarded as a structural property of the score rather than independent biological validation. By contrast, the number of genes with mitochondrial gene-name prefixes (*NDUF*, *COX*, *UQCR*, *ATP5*, *MRPL*, *MRPS*, *SDH*, *CYC1*, *CHCHD*, *TIMM*, and *TOMM*) in the fast-ICS top 100 (25 vs. a null mean of 1.6, *p* = 0.003) exceeded that expected under the input-permutation null.

#### 2.4.2. Robustness of the Ranking

The ranking was moderately stable to the choice of weights but sensitive to the definition of the methylation gene-level score (Table S2). Across 1000 random weight vectors, the main top 100 retained a median of 67 genes (IQR 56–79). Thirty-four genes stayed in the top 100 under ≥ 80% of weight vectors.

Changing the C3 leading-edge threshold from the top 25% to the top 10% or 50% retained 77 and 66 genes. Using methylation only at the mechanism (set) level retained 33 genes and reduced the mitochondrial count from 44 to 15. The gene-level methylation score therefore shapes the composition of the top genes substantially, including their mitochondrial content.

In the patient bootstrap, twenty of the top 100 genes entered the bootstrap top 100 in ≥80% of resamples and 56 in ≥50%. *MRPL34*, *UQCC1*, and *PROCR* entered in every resample, and *EGR1* in 99.5%. The 95% bootstrap rank intervals of the top 100 genes were wide (median width 789 ranks). Bootstrap inclusion frequencies and rank intervals therefore indicated uncertainty in top-100 membership and in individual ranks, supporting interpretation at the level of modules and mechanisms.

#### 2.4.3. A Context Map Places the Core ICS Modules Within Established Mechanisms

**Figure 6:**
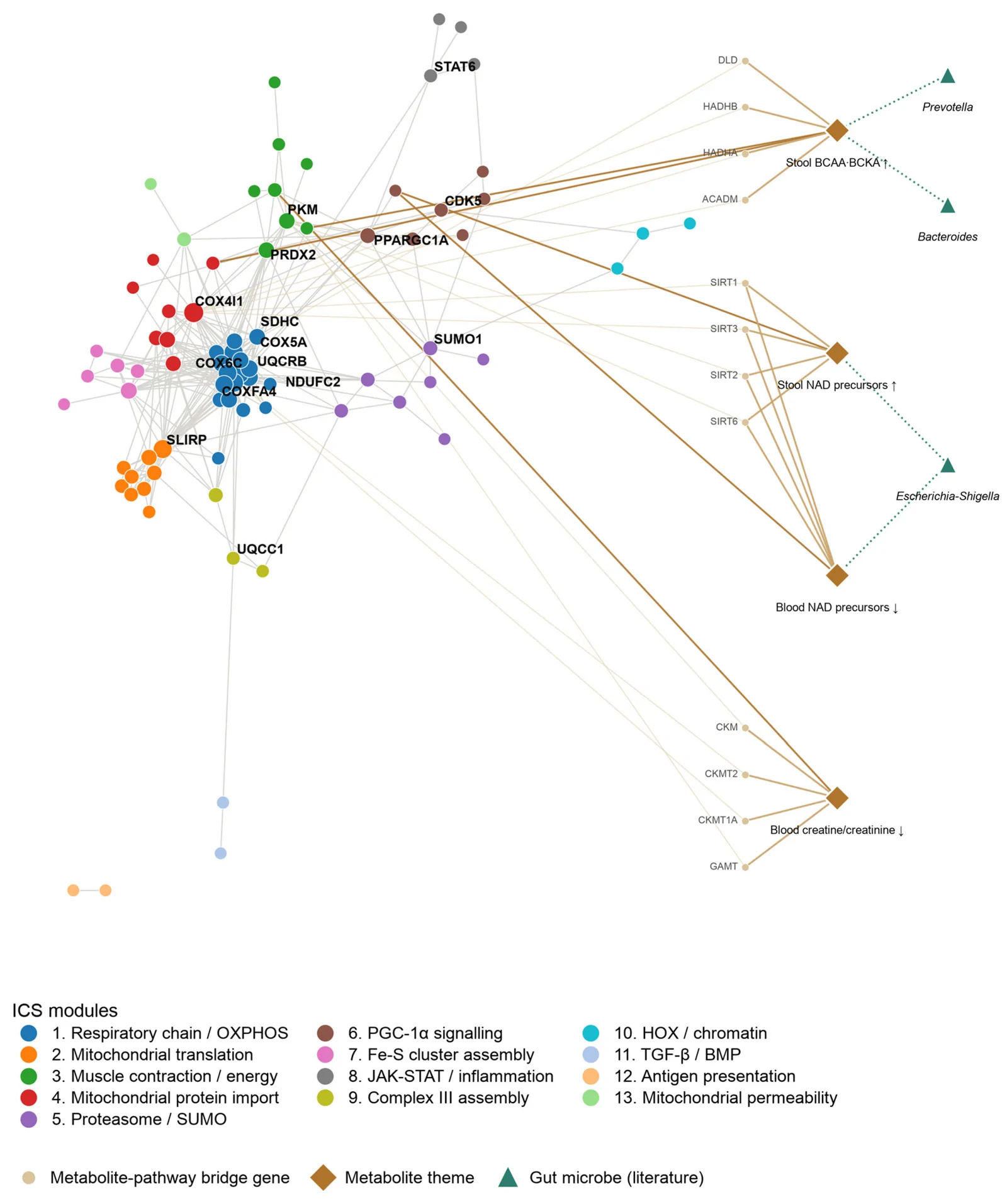
Context map showing the relationship between prior knowledge and our results. STRING network of the ICS top 100 genes (circles; size, degree; colour, module; bold, highest betweenness). Metabolite themes with nominal differences in sarcopenia (diamonds; faecal and blood; ↑, higher and ↓, lower in sarcopenia) are joined to genes by pre-defined pathway membership (dark lines) or through bridge genes outside the top 100 (beige; light lines). Gut microbes (triangles) are linked to themes by published associations only (dotted lines) and are hypotheses. Modules and key nodes are listed in Table S1.

To relate the ICS result to prior knowledge, the ICS top 100 genes were organised into network modules and connected to metabolite themes and gut microbes through pre-defined pathways and the literature (Figure 6).

The 79 connected genes of the top 100 formed 13 modules (Table S1). The largest was the respiratory chain/OXPHOS module (18 genes; hubs *SDHC*, *UQCRB*, *COX6C*, and *COX5A*). It was followed by mitochondrial translation (9 genes; hub *SLIRP*), muscle contraction/energy (8 genes; hubs *PKM*, *PRDX2*, *CKB*, and *TPM2*) and mitochondrial protein import (8 genes; hub *COX4I1*). The proteasome/SUMO (7 genes; hub *SUMO1*) and PGC-1α signalling (7 genes; hub *PPARGC1A*) modules were the next largest.

These modules combine evidence from different layers. For example, the muscle contraction/energy module contained four methylation layer-hit and four GWAS layer-hit genes, and the PGC-1α module five GWAS-hit genes. The ten key nodes by betweenness centrality (among the 15 genes shown in bold in Figure 6) were *COX4I1*, *PKM*, *PPARGC1A*, *PRDX2*, *SLIRP*, *SDHC*, *UQCRB*, *SUMO1*, *CDK5*, and *COX6C*. *COX4I1* had the highest betweenness. *UQCRB* and *COX5A* linked eight modules, and *COX4I1*, *SLIRP*, *PPARGC1A*, *PPIF*, and *COXFA4* seven. *PKM* and *PRDX2* linked the contraction module to the mitochondrial modules.

Metabolite themes provided independent context (Figure S5). In the faecal metabolome, branched-chain amino acids and keto acids (BCAA/BCKA; z = 2.78, *p* = 0.005, FDR = 0.019) and NAD precursors (z = 2.89, *p* = 0.004, FDR = 0.019) were higher in sarcopenia. In blood, NAD precursors (z = −2.53, *p* = 0.011, FDR = 0.10) and creatine/creatinine (z = −2.07, *p* = 0.039, FDR = 0.17) were lower, although neither remained significant after correction for multiple testing.

These themes linked to the ICS modules through pathway membership. For BCAA, *BCKDHB* and *ECHS1* were within the top 100, and *HADHA*, *HADHB*, *ACADM*, and *DLD* acted as bridges to *COX4I1* and *COX5A*. For NAD precursors, *SIRT4* was within the top 100, and *SIRT1*, *SIRT3*, *SIRT2*, and *SIRT6* acted as bridges to *COX4I1* and *PKM*. For creatine/creatinine, *CKB* was within the top 100, and *CKM*, *CKMT1A*, *CKMT2*, and *GAMT* acted as bridges to *PKM*, *COX6C*, and *CKB*.

Gut taxa with published metabolic links to these themes were added as hypotheses: *Prevotella* and *Bacteroides* for BCAA biosynthesis, and *Escherichia-Shigella* for NAD precursor deamidation.

The map is descriptive, and its links are pathway definitions or literature associations, not tested causal paths. Within these limits, it places the core ICS modules within three established axes of muscle ageing: mitochondrial respiration, the creatine/phosphocreatine energy shuttle of contraction, and NAD-dependent PGC-1α signalling.

## 3. Discussion

This study addressed a practical challenge in sarcopenia multi-omics research: integrating heterogeneous cohorts and omics layers that capture different aspects of biology [10]. This study yielded three main findings. First, across the five muscle transcriptome cohorts, few genes showed statistically significant changes, and gene-level findings were not consistent across cohorts, whereas mitochondrial pathway scores were lower in the pooled analysis and in most individual cohorts, and mitochondrial signals were also lower in ageing- and frailty-related comparisons. Second, the transcriptome, ageing methylation, and GWAS layers highlighted different biological processes, and gene-level convergence was not established. Third, integrating these layer-specific mechanisms with network information (the ICS) produced top-ranked genes that were more connected than those from conventional combined statistics, partly by construction, and contained more interactions between genes supported by different layers. A context map placed the resulting modules within established axes of muscle ageing.

The limited gene-level findings are expected given the analytic sample sizes of 23–60 participants per cohort and the differences in sarcopenia definitions, ethnicities, and platforms. The excess of small *p* values was consistent with a distributed signal, although individual gene associations were not robust to the more conservative meta-analysis: six genes reached FDR < 0.05 in the random-effects meta-analysis, and none remained FDR-significant when the Hartung–Knapp–Sidik–Jonkman method, a more conservative approach for meta-analyses of few studies, was applied.

At the pathway level, by contrast, mitochondrial respiration, mitochondrial translation and contraction were lower in the pooled analysis and in most individual cohorts. This agrees with the MEMOSA study, which reported reduced mitochondrial oxidative capacity and NAD^+^ biosynthesis in sarcopenic muscle across ethnicities [9]. By reanalysing the three MEMOSA cohorts alongside two additional cohorts, the present analysis extends that work in three ways. First, participants from five cohorts were placed on a common scale relative to each cohort’s healthy participants. Second, pre-sarcopenia and single-criterion groups were included. Third, in the Jamaican cohort, the direction of the pathway signal depended on whether participants meeting a single impairment criterion were included in the reference group. In these cohorts, pathway-level patterns were therefore more consistent than individual gene-level associations, which motivated building the integration on mechanisms.

The mitochondrial signal was not confined to sarcopenia. The mitochondrial/OXPHOS module was lower with ageing, with frailty, and at pre-sarcopenia at nominal *p* < 0.05 (FDR 0.06–0.07), and the sarcopenia comparison showed the same direction but did not reach statistical significance. Mitochondrial pathway scores were also lower with age in healthy older adults, and the ageing and sarcopenia signatures were most strongly correlated among mitochondrial genes. These observations are consistent with the age-related decline of mitochondrial biogenesis and AMPK activity in muscle [17], with mitochondrial quality-control failure in sarcopenia [18], and with recent single-cell atlases of the ageing human muscle [4,5]. They also agree with our previous cross-species study, which identified reduced complex I activity and dysregulated mitochondrial transcription and translation in sarcopenic muscle [19].

Together, the data are consistent with a mitochondrial component that sarcopenia shares with muscle ageing. Because the comparisons involve different groups and all data are cross-sectional, they do not demonstrate a stepwise progression from ageing to sarcopenia or that ageing causes the decline. The spindle/chromatin module behaved differently: it was higher in the disease comparisons and only slightly higher with age, although a difference between these effects was not formally tested. One possibility is that it reflects altered regenerative or cell-cycle activity in sarcopenic muscle, as described for aged muscle stem cell states [20]; this remains to be tested.

Whereas the transcriptome captured these features of the current muscle state, the methylation and GWAS layers highlighted other processes. Each layer, tested against its own null distribution, highlighted different biological processes. The transcriptome was associated with energy metabolism and contraction. The ageing methylation layer was associated with age-related methylation patterns in inflammation, extracellular matrix, and hypoxia, consistent with earlier methylation studies of ageing muscle [21,22] and with the role of fibro-adipogenic, inflammatory and Wnt-driven niche remodelling in aged muscle [23,24]. The GWAS layer was associated with predisposition, dominated by skeletal development and body size. Colocalisation analysis supported a shared causal variant with height or body mass index at 9 of the 21 low grip strength loci (PP4 > 0.8), in line with the strong body-size component of muscle-mass and strength genetics [25,26].

Overlap of genes between layers was close to expectation, and mechanism-level overlap beyond expectation was confined mainly to the methylation and GWAS layers, which shared extracellular matrix and TGF-β–related mechanisms. Because the layers differ in sample size, measured material and power, this does not show that they are independent or complementary, but it argues against treating them as replicates of a single signal.

Because the layers did not converge on the same genes, we integrated them at the level of mechanisms and networks. The central hypothesis was that integrating layer-specific mechanisms and network information would prioritise more connected and functionally coherent gene sets than gene-level score aggregation alone. The conventional comparator was a rank-based, Stouffer-type score aggregation, in which within-layer ranks were converted to normal scores and combined; this weighted-Z approach is transparent and well characterised [16,27]. Against it, the ICS top genes were more connected at every list size and more cohesive from the top 50 onwards, and they contained more interactions between genes supported by different layers, for example, *PPIF*–*CKB*, *PPIF*–*PPARGC1A*, and *UQCC1*–*UQCC2*.

This comparison has several caveats. Part of the network advantage is built in, because the ICS includes a network component. The proportion of cross-layer interactions was similar between the rankings, so the ICS advantage was in the number of such interactions, not their share. In the input-permutation analysis, the network structure of the top 100 did not differ significantly from the null (network cohesion, *p* = 0.062; connected genes, *p* = 0.051). By contrast, the number of genes with mitochondrial gene-name prefixes in the top 100 exceeded that expected under the input-permutation null (*p* = 0.003). Finally, when participants were resampled 200 times, a core of genes (for example, *MRPL34*, *UQCC1* and *PROCR*) remained in the top 100 in every resample, but the exact order of similarly ranked genes changed between resamples; the ranking was also moderately sensitive to the weights and strongly dependent on the gene-level methylation score. We therefore interpret the ICS output at the level of modules and mechanisms rather than individual ranks, and we regard the ICS not as a replacement for statistical combination but as a way of organising different layers into a connected gene network.

At the module level, the context map organised the ICS top 100 into modules for respiratory chain, mitochondrial translation and protein import, muscle contraction and energy, proteasome/SUMO and PGC-1α signalling. *COX4I1* had the highest betweenness, and *PPARGC1A* linked 7 of the 13 modules. PGC-1α is a master regulator of mitochondrial biogenesis, oxidative metabolism, and ROS defence [28], so its position as a connector between modules is biologically plausible.

Three metabolite themes linked to these modules through pre-defined pathways. Each theme was scored as the mean of standardised (z-scored) values of its member metabolites, and multiple testing was controlled separately within the faecal and blood themes. The first theme was NAD precursors. The NAD-precursor theme score was higher in faeces (FDR = 0.019) and nominally lower in blood (*p* = 0.011; FDR = 0.10) in sarcopenia, in separate populations, and the theme connected to *SIRT4* and, through sirtuin bridge genes, to *COX4I1* and *PKM*. Reduced muscle NAD^+^ biosynthesis has been reported in human sarcopenia [9]. In animal models, microbial nicotinic acid improved muscle mitochondrial function through NAD^+^, SIRT1, and PGC-1α [29], and gut bacteria can also feed host NAD synthesis through nicotinamide deamidation [30]. Together, these findings raise the possibility of a gut–NAD–PGC-1α–mitochondria axis.

The second theme was creatine. The blood creatine/creatinine theme score was nominally lower in sarcopenia (*p* = 0.039; FDR = 0.17) and linked to *CKB*, which lies within the top 100. Creatine kinase isoenzymes form the phosphocreatine system of cellular energy buffering [31]. *CKB* shares a module with *PKM* and *PRDX2*, which link the contraction and mitochondrial modules, and its network position suggests a possible link to cellular energy buffering. The third theme was BCAA/BCKA. The faecal BCAA/BCKA theme score was higher in sarcopenia (FDR = 0.019) and linked to *BCKDHB* and *ECHS1*, which lie within the top 100; gut *Prevotella copri* and *Bacteroides vulgatus* contribute to BCAA biosynthesis [32].

These links are consistent with a proposed gut–muscle axis in muscle ageing [33,34], but they are not tested pathways. The metabolome data come from different and small populations, gut microbes were not measured, and the links are pathway definitions or literature associations. The map should therefore be regarded as hypothesis-generating. Clinical trials of urolithin A have reported improvements in selected muscle performance measures and mitochondrial biomarkers [35,36], providing a rationale for further investigation of mitochondrial interventions.

This study has several limitations. All data are cross-sectional and public. Transcriptome cohorts are small, the largest cohort contributes much of the signal, and four of five cohorts include men only. The methylation layer compares older with younger muscle rather than sarcopenia with controls. The two GWAS share UK Biobank participants, and the genomic layer partly reflects body size. With regard to score construction, the C3 leading-edge threshold was chosen after an initial analysis, although alternative thresholds retained most of the top 100 (Table S2). Bootstrap and permutation analyses used a fast approximation of the mechanism component, so they validate the approximate rather than the exact score. The ranking was also sensitive to the definition of the gene-level methylation score. Network metrics depend on STRING, which is biased toward well-studied genes; the degree-matched null reduces but does not remove this bias. The auxiliary data are also limited: the metabolomic analyses involved small, separate populations and were not adjusted for potential confounders, and gut microbe links are literature-based. Finally, no experimental validation was performed. Prospective cohorts with matched muscle, blood, and stool data, and functional studies of the mitochondrial, creatine, and NAD axes, are needed.

## 4. Materials and Methods

### 4.1. Multi-Omics Data Collection

Three primary omics layers were taken from public human data: skeletal muscle transcriptomes, ageing-associated muscle DNA methylation, and genome-wide association studies (GWAS) of muscle mass and strength. The transcriptome layer comprised five skeletal muscle cohorts with sarcopenia status: GSE226151 (China), GSE111016 (Singapore Sarcopenia Study), GSE111006 (Hertfordshire Sarcopenia Study) and GSE111010 (Jamaica Sarcopenia Study), all RNA-seq, and GSE167186 (Canada; 3′-tag bulk RNA-seq). The main analyses included 208 participants (111 healthy, 77 sarcopenia, and 20 pre-sarcopenia); 24 participants meeting a single criterion were analysed separately (Figure S1). Five old-vs-young muscle datasets and a frailty dataset served as ageing and frailty references. The methylation layer was the older vs. young muscle comparison of Turner et al. [22], and the genomic layer the appendicular lean mass GWAS of Pei et al. [25]. Clinical-trait genes came from the low grip strength GWAS of Jones et al. [26] and strict GWAS Catalog associations. Faecal and blood metabolome data and literature-based gut microbe associations were used only for context, not for ranking. Cohort definitions, quality control and data sources are detailed in the Supplementary Methods.

### 4.2. Layer-Specific Statistics and Gene-Level Scores

**Transcriptome.** Each cohort was analysed from raw counts with PyDESeq2 v0.5.4 [37] (DESeq2, [38]), and cohort estimates were combined by random-effects meta-analysis [39]. For participant-level analyses, each gene was expressed as a z-score relative to the healthy participants of the same cohort:

(1) $$z_{\mathrm{gi}}\;=\;\frac{x_{\mathrm{gi}}\;-\;{\bar{x}}_{g,\mathrm{healthy}}}{s_{g,\mathrm{within}}}$$

where *s_g_*, within is the pooled within-group standard deviation. A participant’s pathway score was the mean z of the member genes, and pathway effects were estimated by OLS with a cohort fixed effect and age. Gene-level transcriptome evidence was the sarcopenia t statistic from OLS of the gene z-score on sarcopenia, age, sex, and cohort. Consensus co-expression modules (22 modules) were built by WGCNA [40] without disease labels and related to ageing, frailty, pre-sarcopenia and sarcopenia (Supplementary Methods).

**Methylation.** A gene carrying at least one FDR-significant differentially methylated CpG (DMC gene; *h_g_* = 1) was scored against its probability of doing so given its number of CpGs, estimated by logistic regression on log CpG count (natural cubic spline), following missMethyl gometh [41]:

(2) $$m_{g}\;=\;\frac{h_{g}\;-\;{\hat{p}}_{g}}{\sqrt{{\hat{p}}_{g}(1\;-\;{\hat{p}}_{g})}}$$

**GWAS.** Variants were mapped to genes with MAGMA v1.10 [42], and lean mass gene z statistics were rank-inverse-normal transformed. Clinical-trait genes were the 28 low grip strength genes passing Bonferroni correction in MAGMA and 19 GWAS Catalog genes. Colocalisation is described in the Supplementary Methods. Effects were not combined across layers as signed values; direction was used only within the transcriptome layer.

### 4.3. Layer-Specific Mechanism Analysis

Mechanisms (MSigDB Hallmark [43], KEGG, Reactome and GO Biological Process sets, all accessed on 21 August 2026, and the 22 modules) were tested in each layer against a null preserving that layer’s dependence structure: for the transcriptome, the competitive set score (mean gene t in the set minus the genome-wide mean) against 4000 within-cohort label permutations; for methylation, DMC-gene over-representation against 4000 random gene sets matched on CpG-count decile; and for GWAS, MAGMA competitive gene-set analysis of lean mass. FDR was controlled within each layer and library. Leading-edge genes of a mechanism at FDR < 0.05 were its members in the top 25% of the layer, in the direction of the set.

### 4.4. Integrated Convergence Score-Based Prioritisation

The ICS was computed for 17,613 genes expressed in skeletal muscle and 11 clinical-trait GWAS genes not expressed in muscle, as the equally weighted mean of four components scaled to 0–1:

ICS*_g_* = 0.25 × (C1*_g_* + C2*_g_* + C3*_g_* + C4*_g_*) (3)

**C1, layer evidence.** For each layer, genes were ranked by their evidence (transcriptome |t|, methylation score *m_g_* and lean mass gene z), and a layer hit was a gene in the top 10% of that layer. With *B_g_* the mean of the three layer percentiles (missing values set to 0.5) and *n_g_* the number of layer hits:

(4) $${C1}_{g}\;=\;\frac{B_{g}\;\times \;\max(1,\;n_{g})}{3}$$

**C2, clinical trait.** C2*_g_* = 1 for the 47 genes associated by GWAS with the clinical diagnostic traits of sarcopenia (28 low grip strength MAGMA genes and 19 GWAS Catalog genes) and 1/3 otherwise.

**C3, mechanism.** With *l_g_* the number of layers in which the gene is a leading-edge member of an FDR < 0.05 mechanism:

(5) $${C3}_{g}\;=\;\frac{l_{g}}{3}$$

**C4, network.** For each gene, the number of STRING v12.5 [44] interaction partners (combined score ≥ 0.7 with experimental or database evidence) within a candidate pool (genes with at least one layer hit and the C2 genes) was compared with the number expected from its total degree by a hypergeometric upper-tail test, and C4*_g_* was the percentile of −log10 P across genes. Genes in the same GWAS locus were additionally summarised by the best-ranked gene per locus.

### 4.5. Conventional Comparator and Network Evaluation

The ICS was compared with a rank-based, Stouffer-type combination of the same layer evidence. Within each layer, gene evidence was rank-inverse-normal transformed (*z_lg_*), and the layer scores were combined as:

(6) $$Z_{g}\;=\;\frac{\sum_{l}z_{\mathrm{lg}}}{\sqrt{k_{g}}}$$

where *k_g_* is the number of layers with a value for gene g (weighted Z-method) [16]. For the top 25–400 genes of each ranking, network cohesion was the number of STRING interactions among the genes divided by the mean for 1000 random gene sets matched on STRING-degree decile, and connectivity was the percentage of genes interacting with another top-N gene. Modules were defined by k-means clustering of random-walk-with-restart profiles. Robustness and replication analyses (leave-one-component-out, random weights, input permutation, participant bootstrap, and leave-one-cohort-out replication) are described in the Supplementary Methods.

### 4.6. Context Map and Statistical Analysis

The ICS top 100 genes were placed in a STRING network and organised into modules, which were linked to pre-defined faecal and blood metabolite themes through pathway membership and to gut microbes through published metabolic associations [30,32]; the map is hypothesis-generating (Supplementary Methods). Tests were two-sided unless stated otherwise, and multiple testing was controlled by the Benjamini–Hochberg procedure [45]. Analyses used Python 3.11 and MAGMA v1.10.

## 5. Conclusions

In the pooled analysis of the five cohorts, sarcopenic muscle showed lower mitochondrial pathway scores, and mitochondrial signals were also lower in ageing- and frailty-related comparisons. Transcriptome, methylation and GWAS layers captured different aspects of the disease: muscle state, age-related methylation patterns, and predisposition. Integrating their mechanisms with network information produced a more connected set of top-ranked genes than conventional combined statistics, which partly reflects the network component of the score. The resulting modules link mitochondrial respiration, creatine-based energy buffering, and NAD–PGC-1α signalling. These computationally prioritised candidates and mechanisms require experimental validation.

## Data Availability

All datasets analysed in this study are publicly available. Accession numbers and sources are listed in Figure S1 and Section 4.1: GEO GSE226151, GSE111016, GSE111006, GSE111010, GSE167186, GSE25941, GSE38718, GSE9103, GSE8479, GSE136344, and GSE117525; GWAS Catalog GCST90000025 and GCST90007526; Metabolomics Workbench ST003002; MetaboLights MTBLS3341; STRING version 12.5; GTEx/eQTL Catalogue QTD000281 and QTD000085; UK Biobank height and body mass index GWAS (Neale laboratory, phenotypes 50 and 21001); the differentially methylated CpG list reported in the supplementary data of reference [22]. The analysis code is available from the corresponding author upon reasonable request, and the code will be made publicly available once intellectual property and licencing procedures are complete.

## Supplementary Materials

The following supporting information can be downloaded at: https://www.mdpi.com/article/10.3390/ijms27219500/s1.

## Abbreviations

The following abbreviations are used in this manuscript:

AMPK	AMP-activated protein kinase
BCAA	branched-chain amino acid
BCKA	branched-chain keto acid
CpG	cytosine–phosphate–guanine dinucleotide
DMC	differentially methylated CpG
ECM	extracellular matrix
EMT	epithelial–mesenchymal transition
eQTL	expression quantitative trait locus
FDR	false discovery rate
GO	Gene Ontology
GPCR	G-protein-coupled receptor
GTEx	Genotype-Tissue Expression
GWAS	genome-wide association study
ICS	integrated convergence score
IQR	interquartile range
KEGG	Kyoto Encyclopedia of Genes and Genomes
MAGMA	Multi-marker Analysis of GenoMic Annotation
MEMOSA	Multi-Ethnic Molecular Determinants of Human Sarcopenia
MSigDB	Molecular Signatures Database
NAD	nicotinamide adenine dinucleotide
NF-κB	nuclear factor kappa B
OLS	ordinary least squares
OXPHOS	oxidative phosphorylation
PGC-1α	peroxisome proliferator-activated receptor gamma coactivator 1-alpha
PP4	posterior probability of a shared causal variant
ROS	reactive oxygen species
SD	standard deviation
STRING	Search Tool for the Retrieval of Interacting Genes/Proteins
TCA	tricarboxylic acid
TNF	tumour necrosis factor
WGCNA	weighted gene co-expression network analysis
