# Supplementary material for: Integrated Multi-Omics Convergence Scoring Reveals Modular Network Architecture with Age-Associated Mitochondrial Signatures in Sarcopenia
Source: Int J Mol Sci. 2026 Oct 24;27(21):9500. doi: 10.3390/ijms27219500 (PMC13762844; doi:10.3390/ijms27219500)
Supplement: Supplementary file 1 [file ijms-27-09500-s001.zip › ijms-4583179-supplementary/Supplementary_Materials_v6.pdf]

# Supplementary Materials

Integrated Multi-Omics Convergence Scoring Reveals Modular Network Architecture with Age-Associated Mitochondrial Signatures in Sarcopenia

## Contents

Figure S1. Sarcopenia transcriptome cohorts and reference datasets.

Figure S2. Leave-one-cohort-out (LOCO) replication of the ICS, combined statistics and the transcriptome-only ranking.

Figure S3. Network structure of the top-ranked genes after removing each ICS component.

Figure S4. Input permutation control for the ICS top 100.

Figure S5. Pre-defined metabolite themes in sarcopenia.

Table S1. Gene sets, co-expression modules and context-map tables.

Table S2. ICS ranking and comparison with combined statistics.

Supplementary Methods (S1–S6)

| Cohort | Population | Platform | Sex | Healthy | Sarcopenia | Pre-sarc. | Partial* | Age, median (range) |
|---|---|---|---|---|---|---|---|---|
| GSE226151 | China | RNA-seq | men and women | 20 | 20 | 20 | 0 | 70 (53–88) |
| GSE111016 | Singapore (SSS) | RNA-seq | men | 20 | 20 | 0 | 0 | 71 (65–79) |
| GSE111006 | UK, Hertfordshire (HSS) | RNA-seq | men | 28 | 4 | 0 | 8 | 72 (68–77) |
| GSE111010 | Jamaica (JSS) | RNA-seq | men | 14 | 9 | 0 | 16 | 75 (63–89) |
| GSE167186 | Canada | 3′-tag RNA-seq | men | 29 | 24 | 0 | 0 | 73 (64–92) |
| **Total** | | | | **111** | **77** | **20** | **24** | |

* Low muscle mass only or low strength only (MEMOSA cohorts). Reference datasets: ageing (old vs young muscle arrays) GSE25941, GSE38718, GSE9103, GSE8479, GSE136344; frailty GSE117525; methylation Turner et al. 2020; GWAS Pei et al. 2020 (ALM), Jones et al. 2021 (low grip), GWAS Catalog; metabolome Metabolomics Workbench ST003002 (faecal), MetaboLights MTBLS3341 (blood).

**Figure S1. Sarcopenia transcriptome cohorts and reference datasets.**

Patient-level counts used in the analysis (208 participants: 111 healthy, 77 sarcopenia, 20 pre-sarcopenia). Partial, low muscle mass only or low strength only (MEMOSA cohorts; analysed separately, not as controls). Age is given as median (range). Reference datasets: ageing (old vs young muscle arrays) GSE25941, GSE38718, GSE9103, GSE8479 and GSE136344; frailty GSE117525 (also contributing a young-vs-old contrast to the module-level ageing meta-analysis); methylation, Turner et al. 2020; GWAS, Pei et al. 2020 (appendicular lean mass), Jones et al. 2021 (low grip strength) and the GWAS Catalog; metabolome, Metabolomics Workbench ST003002 (faecal) and MetaboLights MTBLS3341 (blood).

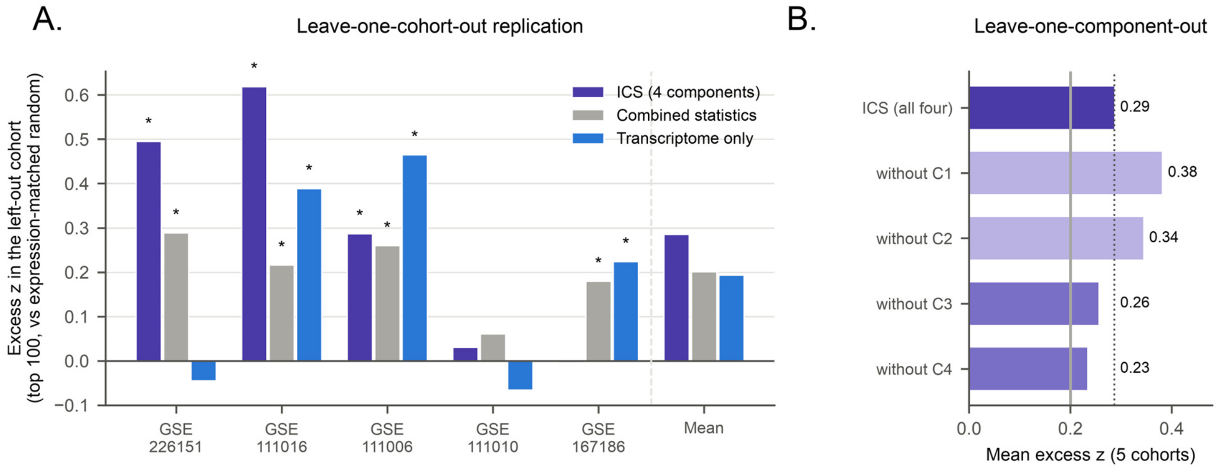


**Figure S2. Leave-one-cohort-out (LOCO) replication of the ICS, combined statistics and the transcriptome-only ranking.**

(A) Each cohort was left out in turn; rankings were rebuilt from the remaining cohorts (fast implementation of the four-component ICS, rank-based Stouffer combined statistics, transcriptome only), and the top 100 genes were evaluated in the left-out cohort's differential-expression statistics (DESeq2). Excess z is the mean direction-aligned z of the top 100 minus that of expression-matched random gene sets; * $P < 0.05$. Pre-specified criterion: ICS at least as good as the transcriptome-only ranking in ≥ 3 of 5 cohorts, which was met (3/5). Mean excess z: ICS 0.29, combined statistics 0.20, transcriptome only 0.19 (direction-aligned mean z, ICS 0.47 vs transcriptome only 0.38); the ICS exceeded combined statistics in 3 of 5 cohorts (mean paired difference 0.08). Without the co-expression-module gene sets, the criterion was also met (3/5; mean excess z 0.33). The transcriptome-only ranking failed to replicate in GSE226151 and GSE111010; the ICS replicated in GSE226151 but not in GSE111010 or GSE167186. (B) Mean excess z across the five cohorts after removing each ICS component (dotted line, full ICS; grey line, combined statistics). Removing the mechanism (C3, 0.26) or network (C4, 0.23) component lowered replication, whereas removing layer evidence (C1, 0.38) or clinical trait (C2, 0.34) did not. Single-component results are given in Table S2.

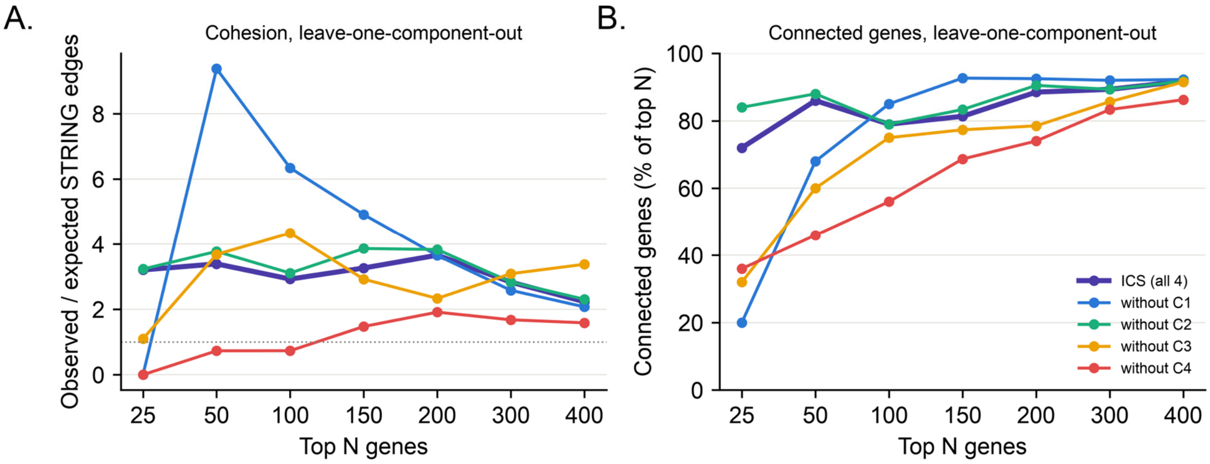


**Figure S3. Network structure of the top-ranked genes after removing each ICS component.**

(A) Network cohesion (observed STRING edges, experiments/databases score ≥ 0.7, divided by the mean of 1,000 random gene sets matched on STRING degree decile; dotted line, 1) and (B) percentage of the top N genes connected to at least one other top-N gene (STRING combined score ≥ 0.4) for the full ICS and after removing each component, computed as in Figure 5A,B. Random sets were redrawn, so full-ICS cohesion differs slightly from Figure 5A (e.g., top 25: 3.21 vs 2.97). Removing the network component (C4) lowered cohesion (top 100: 0.73 times the degree-matched expectation) and connectivity at every size, showing that part of the ICS network advantage is built in through C4. Removing C3 lowered connectivity among the top 25 (32% vs 72%). Without C1, the top 50–150 were dominated by co-regulated respiratory-chain subunits, which inflates cohesion.

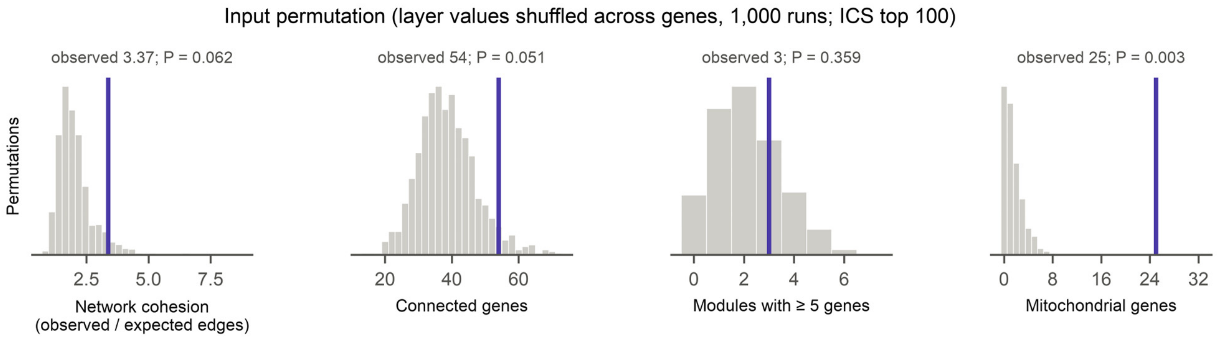


**Figure S4. Input permutation control for the ICS top 100.**

Layer values (transcriptome, methylation and GWAS gene scores) were shuffled across genes 1,000 times and the four-component ICS was recomputed (fast implementation). Histograms show the null distributions for the top 100; purple lines show the observed values. P = (number of permutations ≥ observed + 1)/1,001. Values refer to the top 100 of the fast ICS, which differs slightly from the main ranking (Figure 5). Cohesion 3.37 vs a null mean of 2.06 (P = 0.062); connected genes 54 vs 38.1 (P = 0.051); modules with ≥ 5 genes 3 vs 2.1 (P = 0.36); mitochondrial genes 25 vs 1.6 (P = 0.003). Shuffling GWAS gene labels as well (500 permutations) gave similar results (Table S2). The network structure did not differ significantly from the permutation null, whereas the mitochondrial content of the top 100 was not reproduced by shuffled inputs.

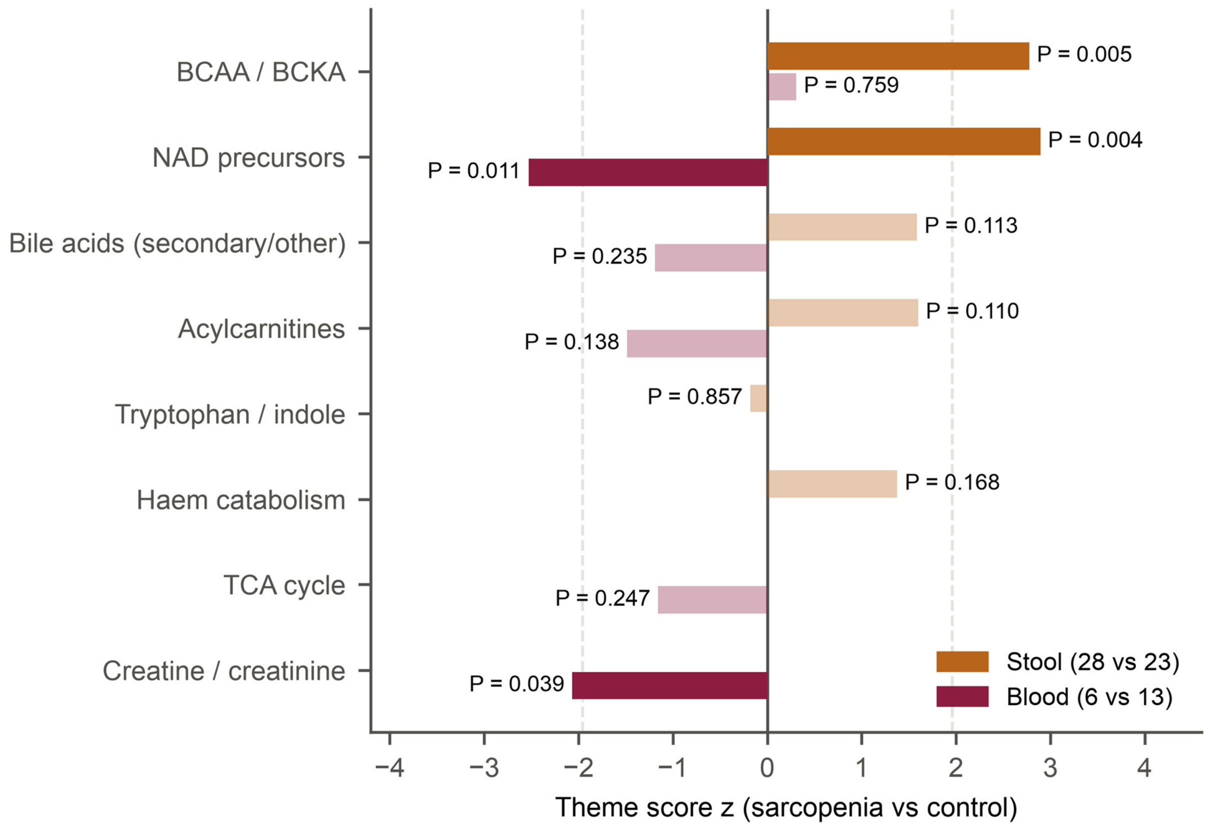


**Figure S5. Pre-defined metabolite themes in sarcopenia.**

Theme scores (mean z of member metabolites, sarcopenia vs control; P from label permutation) in stool (Metabolomics Workbench ST003002; 28 sarcopenia vs 23 controls) and blood (MetaboLights MTBLS3341; 6 vs 13). Full colour, $P < 0.05$ (nominal); pale, $P \geq 0.05$; dashed lines, $|z| = 1.96$. Themes with $P < 0.05$ (stool BCAA/BCKA and NAD precursors, FDR within layer 0.019; blood NAD precursors and creatine/creatinine, FDR 0.10 and 0.17) are the metabolite nodes of the context map in Figure 6. Gut microbes in Figure 6 are linked to these themes only through published metabolic associations (Pedersen et al. 2016; Shats et al. 2020). Values are given in Table S1 (sheet metabolite_themes).

## Table S1. Gene sets, co-expression modules and context-map tables.

Provided as an Excel file (Table_S1_gene_sets_and_modules.xlsx). Sheets: module summary (identifier, functional name, number of genes and naming basis), module gene lists, module over-representation results, official gene-set names and gene lists for every mechanism shown in Figures 2–4, the KEGG human-disease terms collapsed in Figure 4A, the context-map modules, key nodes and links of Figure 6, and the metabolite theme statistics of Figure S5.

### Module summary

| Module | Functional name | Genes |
|---|---|---|
| M1 | Transcription / RNA Pol II | 1750 |
| M2 | Mitochondria / OXPHOS | 1740 |
| M3 | Mito metabolism / proteins | 1313 |
| M4 | ECM / EMT (FAP, immune, adipocyte) | 1294 |
| M5 | GPCR signalling | 1187 |
| M6 | (unlabelled) | 889 |
| M7 | (unlabelled) | 781 |
| M8 | (unlabelled) | 668 |
| M9 | Golgi / vesicle | 635 |
| M10 | Cytosolic translation | 603 |
| M11 | EMT / haemostasis | 413 |
| M12 | RNA processing | 376 |
| M13 | Transcription / chromatin | 360 |
| M14 | Haemostasis / apical junction | 253 |
| M15 | HSP90 / striated contraction | 218 |
| M16 | (unlabelled) | 181 |
| M17 | Transcription / chromatin | 159 |
| M18 | Spindle / chromatin | 108 |
| M19 | Sulfur amino acid | 78 |
| M20 | Cell migration / cardiac AP | 70 |
| M21 | Muscle contraction (type II) | 54 |
| M22 | p53 / mRNA decay | 37 |

### Context-map modules (Figure 6)

| Module | Name | Genes | Metabolites linked | Hub genes |
|---|---|---|---|---|
| 1 | Respiratory chain / OXPHOS | 18 | | SDHC, UQCRB, COX6C, COX5A |
| 2 | Mitochondrial translation | 9 | | SLIRP, MRPS2, MRPS33, MRPL37 |
| 3 | Muscle contraction / energy | 8 | Blood creatine/creatinine ↓, Stool BCAA·BCKA ↑ | PKM, PRDX2, CKB, TPM2 |
| 4 | Mitochondrial protein import | 8 | Stool BCAA·BCKA ↑ | COX4I1, ROMO1, COX14, TIMM13 |
| 5 | Proteasome / SUMO | 7 | | SUMO1, NEDD8, POMP, PSMB3 |
| 6 | PGC-1α signalling | 7 | Blood NAD precursors ↓, Stool NAD precursors ↑ | PPARGC1A, CDK5, STUB1, EGR1 |
| 7 | Fe-S cluster assembly | 6 | | CHCHD2, C1QBP, CHCHD10, BOLA3 |
| 8 | JAK-STAT / inflammation | 4 | | STAT6, DUSP1, PKD1, STING1 |
| 9 | Complex III assembly | 3 | | UQCC1, UQCC2, EIF6 |
| 10 | HOX / chromatin | 3 | | H2AC6, PCGF2, HOXB4 |
| 11 | TGF-β / BMP | 2 | | BMP2, FST |
| 12 | Antigen presentation | 2 | | AP1S1, HLA-A |
| 13 | Mitochondrial permeability | 2 | | PPIF, BAK1 |

**Table S2. ICS ranking and comparison with combined statistics.**

Provided as an Excel file (Table_S2_ICS_ranking_and_comparison.xlsx). Sheets: ICS_ranking, full ranking of all 17,624 scored genes with the ICS, its four components (C1 layer evidence, C2 clinical trait, C3 mechanism, C4 network), layer hits (top 10% of each layer), leading-edge layers, layer statistics (transcriptome t, methylation score, lean mass gene z), clinical-trait GWAS source and locus, STRING degree, rank and Z of the rank-based Stouffer combined statistics, and co-expression module; (a) leave-one-component-out, single components and alternative C2 values; (b) Dirichlet random weights (1,000 draws), per-gene top-100 frequency; (c) C3 leading-edge threshold (top 10%, 25% [main], 50%); (d) methylation gene-level score vs set-level use only; (e) input permutation (1,000 runs; 500 also shuffling GWAS labels); (f) patient bootstrap (200 resamples within cohort × group strata): median rank, 95% interval and top-100 frequency; (g) leave-one-cohort-out results for the ICS, combined statistics and the transcriptome-only ranking; (h) leave-one-cohort-out with each component removed or used alone; (i) STRING interactions linking genes supported by different layers among the top 25, 50 and 100 genes (post hoc, descriptive).

**(a) Leave-one-component-out and single components (genes retained from the main top 10 and top 100)**

| Variant | Spearman ϱ vs main | Top 10 kept | Top 100 kept | GWAS genes in top 100 | Mitochondrial genes in top 100 |
|---|---|---|---|---|---|
| main (0.25 each, C1-C4) | 1.0 | 10 | 100 | 9 | 44 |
| drop C1 | 0.956 | 5 | 53 | 14 | 64 |
| drop C2 | 0.998 | 7 | 91 | 0 | 46 |
| drop C3 | 0.941 | 5 | 59 | 23 | 44 |
| drop C4 | 0.61 | 9 | 59 | 10 | 24 |
| only C1 | 0.446 | 2 | 31 | 1 | 26 |
| only C2 | 1.0 | 3 | 62 | 47 | 31 |
| only C3 | 0.974 | 5 | 93 | 2 | 43 |
| only C4 | 0.93 | 0 | 13 | 0 | 98 |
| simple B (3-layer mean pct) | 0.444 | 4 | 30 | 1 | 25 |
| C2 non-specific = 0.0 | 1.0 | 8 | 88 | 21 | 43 |
| C2 non-specific = 0.5 | 1.0 | 9 | 94 | 3 | 45 |

**(c) C3 leading-edge threshold**

| Leading-edge threshold | Spearman ϱ vs main | Top 10 kept | Top 50 kept | Top 100 kept | GWAS genes in top 100 | Mitochondrial genes in top 100 |
|---|---|---|---|---|---|---|
| 10% | 0.965 | 8 | 31 | 77 | 12 | 48 |
| 25% | 1.0 | 10 | 50 | 100 | 9 | 44 |
| 50% | 0.954 | 7 | 37 | 66 | 6 | 37 |

**(d) Methylation gene-level score**

| Variant | Spearman ϱ vs main | Top 10 kept | Top 50 kept | Top 100 kept | Mitochondrial genes in top 100 | GWAS genes in top 100 |
|---|---|---|---|---|---|---|
| main (CpG-residual meth z) | 1.0 | 10 | 50 | 100 | 44 | 9 |
| C1 without gene-level meth | 0.985 | 3 | 27 | 52 | 28 | 12 |
| C3 without meth leading-edge | 0.983 | 6 | 29 | 64 | 36 | 16 |
| both (meth at set level only) | 0.955 | 1 | 13 | 33 | 15 | 17 |

### (h) Leave-one-cohort-out by component (mean excess z over five cohorts)

| Ranking | Mean excess z | Cohorts with P < 0.05 | Cohorts ≥ full ICS |
|---|---|---|---|
| C4 only | 0.5796 | 2 | 2 |
| without C1 | 0.3804 | 3 | 4 |
| without C2 | 0.3434 | 3 | 3 |
| C1 only | 0.3048 | 4 | 3 |
| ICS (all four) | 0.2862 | 3 | 5 |
| without C3 | 0.2554 | 3 | 2 |
| without C4 | 0.2332 | 3 | 1 |
| C3 only | 0.213 | 2 | 1 |
| Combined statistics | 0.2002 | 3 | 2 |
| Transcriptome only | 0.1906 | 3 | 2 |
| C2 only | 0.0232 | 1 | 1 |

### (i) Cross-layer STRING interactions among top-ranked genes (post hoc)

| Ranking | top | edges | cross_layer_edges | cross_layer_pct | genes_in_cross_layer_edges | genes_with_hits_in_2plus_layers |
|---|---|---|---|---|---|---|
| ICS (4 components) | 25 | 21 | 11 | 52.4 | 13 | 23 |
| ICS (4 components) | 50 | 114 | 43 | 37.7 | 29 | 41 |
| ICS (4 components) | 100 | 330 | 113 | 34.2 | 61 | 81 |
| Combined statistics | 25 | 3 | 1 | 33.3 | 2 | 21 |
| Combined statistics | 50 | 18 | 4 | 22.2 | 8 | 41 |
| Combined statistics | 100 | 41 | 16 | 39.0 | 26 | 78 |

## Supplementary Methods

### S1. Cohorts, Quality Control and Data Sources

The transcriptome layer comprised five skeletal muscle cohorts with sarcopenia status: GSE226151 (China), GSE111016 (Singapore Sarcopenia Study), GSE111006 (Hertfordshire Sarcopenia Study) and GSE111010 (Jamaica Sarcopenia Study), all RNA-seq, and GSE167186 (Canada; McMaster University–Buck Institute study; 3′-tag bulk RNA-seq). Sarcopenia was defined as in each original study. In GSE111006 and GSE111010, where the individual criteria were available, controls met none of the criteria, and the 24 participants meeting only the low-mass or only the low-strength criterion were analysed as separate groups (Figure S1). One GSE111016 sample with a low read-assignment rate (GSM3020407) was excluded from gene-level analyses; excluding it from pathway analyses changed estimates by < 0.01. Five old-vs-young muscle array datasets (GSE25941, GSE38718, GSE9103, GSE8479 and GSE136344) and a frailty dataset with young and older controls (GSE117525) served as ageing and frailty references.

The methylation layer was the genome-wide comparison of older vs young skeletal muscle on the Illumina EPIC array [1]. The genomic layer used full summary statistics of the UK Biobank appendicular lean mass GWAS (GCST90000025) [2]. Clinical-trait genes were taken from the low grip strength GWAS of adults aged ≥ 60 years defined by EWGSOP thresholds (GCST90007526) [3] and from GWAS Catalog associations ($P < 5 \times 10^{-8}$; sarcopenia, grip strength, appendicular lean or skeletal muscle mass and skeletal muscle index), excluding studies whose participants overlap those of the two GWAS above. UK Biobank height and body mass index GWAS (Neale laboratory; phenotypes 50 and 21001) were used for colocalisation. Faecal (Metabolomics Workbench ST003002; 28 sarcopenia vs 23 controls) and blood (MetaboLights MTBLS3341; 6 vs 13) metabolome data were analysed as auxiliary evidence. Genes were mapped to HGNC-approved symbols.

### S2. Differential Expression and Pathway Scores

Each cohort was analysed from raw counts with PyDESeq2 v0.5.4 [4,5] using the Wald test, a common expression filter (count ≥ 10 in at least max(3, 20% of samples)) and within-cohort standardised age as a covariate, plus sex for GSE226151 (the MEMOSA cohorts include men only; sex was unavailable for GSE167186). Cohort log2 fold changes were combined by DerSimonian–Laird random-effects meta-analysis [6] for genes measured in at least three cohorts (17,712 genes), with Hartung–Knapp–Sidik–Jonkman P values [7,8] as a small-sample sensitivity analysis.

For participant-level analyses, counts were normalised within each cohort (median-of-ratios size factors; log2[normalised count + 1]) before z-scoring against the cohort's healthy participants. Pathway scores required at least 10 measured member genes and were computed for all MSigDB Hallmark [9] and KEGG 2021 [10] gene sets and for five selected Reactome [11] and GO Biological Process 2023 [12] sets (complex I biogenesis, mitochondrial translation and cilium assembly; sarcomere organisation and extracellular matrix organisation). The mechanism analyses used all Reactome and GO Biological Process sets of 15–500 genes. The methylation score used CpG-to-gene assignment from the EPIC manifest annotation (UCSC_RefGene_Name) and a natural cubic spline with 5 degrees of freedom [13].

### S3. Co-Expression Modules and Ageing Comparisons

Consensus co-expression modules were constructed by WGCNA [14] in the four full-length RNA-seq cohorts without disease labels (signed biweight-midcorrelation networks, soft threshold at signed scale-free fit $R^2 \geq 0.80$, consensus topological overlap, dynamic tree cutting with minimum module size 30, merging at eigengene correlation ≥ 0.75). This gave 22 modules, all preserved in GSE167186; seven outlier samples identified during network construction were excluded from the module analyses. Module eigengenes were related to ageing (random-effects meta-analysis of six old-vs-young contrasts: the five ageing datasets and the young and older participants of GSE117525), frailty, pre-sarcopenia and sarcopenia. Agreement between the ageing and sarcopenia meta-analysis signatures was summarised by Spearman's ρ, overall and for MitoCarta 3.0 mitochondrial genes [15]. Sensitivity analyses used the original control definition of GSE111010 and men only.

## S4. Gene Mapping and Colocalisation

Variants were mapped to genes with MAGMA v1.10 [16] using a window of 35 kb upstream and 10 kb downstream (NCBI build 37.3), the 1000 Genomes European reference and the SNP-wise mean model. Low grip strength genes were those passing Bonferroni correction outside the MHC region (28 genes); GWAS Catalog genes were taken from the catalogue's positional mapping (19 protein-coding genes). Colocalisation of the 21 low grip strength loci with skeletal muscle eQTL (GTEx via the eQTL Catalogue [17]; FUSION as replication) and with height and body mass index was tested with coloc.abf [18] ($p1 = p2 = 10^{-4}$, $p12 = 10^{-5}$; ±500 kb; PP4 > 0.8). Cross-layer overlap of nominally significant mechanisms was compared with a permutation null.

## S5. Network Evaluation, Replication and Robustness

Network cohesion used the same local STRING v12.5 [19] interactions as C4 (combined score ≥ 0.7 with experimental or database evidence), whereas connectivity, module maps and cross-layer interactions were obtained through the STRING application programming interface (combined score ≥ 0.4). Modules were defined by k-means clustering of random-walk-with-restart profiles [20], with the number of clusters maximising Newman modularity. For the top 25 and top 100, genes in the top 10% of each layer and interactions between genes supported by different layers were counted. A transcriptome-only ranking ($|t|$) was used as a further reference.

In a pre-specified leave-one-cohort-out analysis, all cohort-dependent inputs (transcriptome evidence, layer hits, leading-edge genes, C4 candidate pool and gene directions) were recomputed without each cohort, and the top 100 genes of each ranking were evaluated in the left-out cohort as the direction-aligned mean z (log2 fold change/SE) minus that of 1,000 expression-matched random gene sets (excess z). The criterion was that the ICS be at least as good as the transcriptome-only ranking in ≥ 3 of 5 cohorts and on average. The analysis was repeated without the module gene sets and with each component removed or used alone (Figure S2; Table S2).

Robustness was examined by removing each component, using each component alone, 1,000 Dirichlet-distributed random weight vectors, C3 leading-edge thresholds of the top 10% and 50%, and using methylation only at the mechanism level. An input-permutation control shuffled the three layers' values across genes 1,000 times and recomputed the score; mitochondrial genes were counted by gene-name prefix (NDUF, COX, UQCR, ATP5, MRPL, MRPS, SDH, CYC1, CHCHD, TIMM and TOMM). Rank uncertainty was estimated by resampling participants 200 times within cohort × group strata. Resampling analyses used a parametric mechanism

component (√n × mean rank-inverse-normal z; FDR < 0.05; top-25% leading edge), with C1, C2 and C4 computed exactly.

### S6. Context Map and Metabolome Analysis

The ICS top 100 genes were placed in a STRING network (combined score ≥ 0.4) and clustered as above; modules were named by over-represented gene sets (hypergeometric test against muscle-expressed genes, FDR < 0.05), and key nodes were defined by betweenness centrality. Metabolite values were log2-transformed (faecal values after adding half the smallest positive value). Pre-defined themes (seven faecal, nine blood) were scored as the per-sample mean z of member metabolites, with P from label permutation (10,000 for faecal samples; all 27,132 assignments for blood) and FDR controlled within each sample type. Themes with nominal differences were linked to genes by pre-defined pathway membership: branched-chain amino acids through KEGG valine, leucine and isoleucine degradation; NAD precursors through KEGG nicotinate and nicotinamide metabolism; and creatine/creatinine through genes of creatine synthesis, transport and phosphorylation (GATM, GAMT, SLC6A8, CKB, CKM, CKMT1A and CKMT2). Interacting pathway genes outside the top 100 were shown as bridge genes, and gut microbes were linked to themes through published metabolic associations [21,22]. The map is hypothesis-generating, not a causal mediation analysis.

Analyses used Python 3.11 (PyDESeq2, statsmodels, SciPy, NetworkX; WGCNA implemented following the published algorithm), MAGMA v1.10 and STRING (v12.5 local file and API), with fixed random seeds.

### References for the Supplementary Methods

1. Turner, D.C.; Gorski, P.P.; Maasar, M.F.; Seaborne, R.A.; Baumert, P.; Brown, A.D.; Kitchen, M.O.; Erskine, R.M.; Dos-Remedios, I.; Voisin, S.; Eynon, N.; Sultanov, R.I.; Borisov, O.V.; Larin, A.K.; Semenova, E.A.; Popov, D.V.; Generozov, E.V.; Stewart, C.E.; Drust, B.; Owens, D.J.; Ahmetov, I.I.; Sharples, A.P. DNA methylation across the genome in aged human skeletal muscle tissue and muscle-derived cells: the role of HOX genes and physical activity. *Sci. Rep.* **2020**, *10*, 15360. https://doi.org/10.1038/s41598-020-72730-z

2. Pei, Y.F.; Liu, Y.Z.; Yang, X.L.; Zhang, H.; Feng, G.J.; Wei, X.T.; Zhang, L. The genetic architecture of appendicular lean mass characterized by association analysis in the UK Biobank study. *Commun. Biol.* **2020**, *3*, 608. https://doi.org/10.1038/s42003-020-01334-0

3. Jones, G.; Trajanoska, K.; Santanasto, A.J.; Stringa, N.; Kuo, C.L.; Atkins, J.L.; Lewis, J.R.; Duong, T.; Hong, S.; Biggs, M.L.; Luan, J.; Sarnowski, C.; Lunetta, K.L.; Tanaka, T.; Wojczynski, M.K.; Cvejkus, R.; Nethander, M.; Ghasemi, S.; Yang, J.; Zillikens, M.C.; Walter, S.; Sicinski, K.; Kague, E.; Ackert-Bicknell, C.L.; Arking, D.E.; Windham, B.G.; Boerwinkle, E.; Grove, M.L.; Graff, M.; Spira, D.; Demuth, I.; van der Velde, N.; de Groot, L.C.P.G.M.; Psaty, B.M.; Odden, M.C.; Fohner, A.E.; Langenberg, C.; Wareham, N.J.; Bandinelli, S.; van Schoor, N.M.; Huisman, M.; Tan, Q.; Zmuda, J.; Mellström, D.; Karlsson, M.; Bennett, D.A.; Buchman, A.S.; De Jager, P.L.; Uitterlinden, A.G.; Völker, U.; Kocher, T.; Teumer, A.; Rodriguéz-Mañas, L.; García, F.J.; Carnicero, J.A.; Herd, P.; Bertram, L.; Ohlsson, C.; Murabito, J.M.; Melzer, D.; Kuchel, G.A.; Ferrucci, L.; Karasik, D.; Rivadeneira, F.; Kiel, D.P.; Pilling, L.C. Genome-wide meta-analysis of muscle weakness identifies 15 susceptibility loci in older men and women. *Nat. Commun.* **2021**, *12*, 654. https://doi.org/10.1038/s41467-021-20918-w

4. Muzellec, B.; Teleńczuk, M.; Cabeli, V.; Andreux, M. PyDESeq2: a python package for bulk RNA-seq differential expression analysis. *Bioinformatics* **2023**, *39*, btad547. https://doi.org/10.1093/bioinformatics/btad547

5. Love, M.I.; Huber, W.; Anders, S. Moderated estimation of fold change and dispersion for RNA-seq data with DESeq2. *Genome Biol.* **2014**, *15*, 550. https://doi.org/10.1186/s13059-014-0550-8

6. DerSimonian, R.; Laird, N. Meta-analysis in clinical trials. *Control. Clin. Trials* **1986**, *7*, 177–188. https://doi.org/10.1016/0197-2456(86)90046-2

7. Hartung, J.; Knapp, G. On tests of the overall treatment effect in meta-analysis with normally distributed responses. *Stat. Med.* **2001**, *20*, 1771–1782. https://doi.org/10.1002/sim.791

8. Sidik, K.; Jonkman, J.N. A simple confidence interval for meta-analysis. *Stat. Med.* **2002**, *21*, 3153–3159. https://doi.org/10.1002/sim.1262

9. Liberzon, A.; Birger, C.; Thorvaldsdóttir, H.; Ghandi, M.; Mesirov, J.P.; Tamayo, P. The Molecular Signatures Database (MSigDB) hallmark gene set collection. *Cell Syst.* **2015**, *1*, 417–425. https://doi.org/10.1016/j.cels.2015.12.004

10. Kanehisa, M.; Goto, S. KEGG: kyoto encyclopedia of genes and genomes. *Nucleic Acids Res.* **2000**, *28*, 27–30. https://doi.org/10.1093/nar/28.1.27

11. Milacic, M.; Beavers, D.; Conley, P.; Gong, C.; Gillespie, M.; Griss, J.; Haw, R.; Jassal, B.; Matthews, L.; May, B.; Petryszak, R.; Ragueneau, E.; Rothfels, K.; Sevilla, C.; Shamovsky, V.; Stephan, R.; Tiwari, K.; Varusai, T.; Weiser, J.; Wright, A.; Wu, G.; Stein, L.; Hermjakob, H.; D'Eustachio, P. The Reactome Pathway Knowledgebase 2024. *Nucleic Acids Res.* **2024**, *52*, D672–D678. https://doi.org/10.1093/nar/gkad1025

12. Gene Ontology Consortium; Aleksander, S.A.; Balhoff, J.; Carbon, S.; Cherry, J.M.; Drabkin, H.J.; Ebert, D.; Feuermann, M.; Gaudet, P.; Harris, N.L.; Hill, D.P.; Lee, R.; Mi, H.; Moxon, S.; Mungall, C.J.; Muruganugan, A.; Mushayahama, T.; Sternberg, P.W.; Thomas, P.D.; Van Auken, K.; Ramsey, J.; Siegele, D.A.; Chisholm, R.L.; Fey, P.; Aspromonte, M.C.; Nugnes, M.V.; Quaglia, F.; Tosatto, S.; Giglio, M.; Nadendla, S.; Antonazzo, G.; Attrill, H.; Dos Santos, G.; Marygold, S.; Strelets, V.; Tabone, C.J.; Thurmond, J.; Zhou, P.; Ahmed, S.H.; Asanitthong, P.; Luna Buitrago, D.; Erdol, M.N.; Gage, M.C.; Ali Kadhum, M.; Li, K.Y.C.; Long, M.; Michalak, A.; Pesala, A.; Pritazahra, A.; Saverimuttu, S.C.C.; Su, R.; Thurlow, K.E.; Lovering, R.C.; Logie, C.; Oliferenko, S.; Blake, J.; Christie, K.; Corbani, L.; Dolan, M.E.; Drabkin, H.J.; Hill, D.P.; Ni, L.; Sitnikov, D.; Smith, C.; Cuzick, A.; Seager, J.; Cooper, L.; Elser, J.; Jaiswal, P.; Gupta, P.; Jaiswal, P.; Naithani, S.; Lera-Ramirez, M.; Rutherford, K.; Wood, V.; De Pons, J.L.; Dwinell, M.R.; Hayman, G.T.; Kaldunski, M.L.; Kwitek, A.E.; Laulederkind, S.J.F.; Tutaj, M.A.; Vedi, M.; Wang, S.J.; D'Eustachio, P.; Aimo, L.; Axelsen, K.; Bridge, A.; Hyka-Nouspikel, N.; Morgat, A.; Aleksander, S.A.; Cherry, J.M.; Engel, S.R.; Karra, K.; Miyasato, S.R.; Nash, R.S.; Skrzypek, M.S.; Weng, S.; Wong, E.D.; Bakker, E.; Berardini, T.Z.; Reiser, L.; Auchincloss, A.; Axelsen, K.; Argoud-Puy, G.; Blatter, M.C.; Boutet, E.; Breuza, L.; Bridge, A.; Casals-Casas, C.; Coudert, E.; Estreicher, A.; Livia Famiglietti, M.; Feuermann, M.; Gos, A.; Gruaz-Gumowski, N.; Hulo, C.; Hyka-Nouspikel, N.; Jungo, F.; Le Mercier, P.; Lieberherr, D.; Masson, P.; Morgat, A.; Pedruzzi, I.; Pourcel, L.; Poux, S.; Rivoire, C.; Sundaram, S.; Bateman, A.; Bowler-Barnett, E.; Bye-A-Jee, H.; Denny, P.; Ignatchenko, A.; Ishtiaq, R.; Lock, A.; Lussi, Y.; Magrane, M.; Martin, M.J.; Orchard, S.; Raposo, P.; Speretta, E.; Tyagi, N.; Warner, K.; Zaru, R.; Diehl, A.D.; Lee, R.; Chan, J.; Diamantakis, S.; Raciti, D.; Zarowiecki, M.; Fisher, M.; James-Zorn, C.; Ponferrada, V.; Zorn, A.; Ramachandran, S.; Ruzicka, L.; Westerfield, M. The Gene Ontology knowledgebase in 2023. *Genetics* **2023**, *224*, iyad031. https://doi.org/10.1093/genetics/iyad031

13. Phipson, B.; Maksimovic, J.; Oshlack, A. missMethyl: an R package for analyzing data from Illumina's HumanMethylation450 platform. *Bioinformatics* **2016**, *32*, 286–288. https://doi.org/10.1093/bioinformatics/btv560

14. Langfelder, P.; Horvath, S. WGCNA: an R package for weighted correlation network analysis. *BMC Bioinformatics* **2008**, *9*, 559. https://doi.org/10.1186/1471-2105-9-559

15. Rath, S.; Sharma, R.; Gupta, R.; Ast, T.; Chan, C.; Durham, T.J.; Goodman, R.P.; Grabarek, Z.; Haas, M.E.; Hung, W.H.W.; Joshi, P.R.; Jourdain, A.A.; Kim, S.H.; Kotrys, A.V.; Lam, S.S.; McCoy, J.G.; Meisel, J.D.; Miranda, M.; Panda, A.; Patgiri, A.; Rogers, R.; Sadre, S.; Shah, H.; Skinner, O.S.; To, T.L.; Walker, M.A.; Wang, H.; Ward, P.S.; Wengrod, J.; Yuan, C.C.; Calvo, S.E.; Mootha, V.K. MitoCarta3.0: an updated mitochondrial proteome now with sub-organelle localization and pathway annotations. *Nucleic Acids Res.* **2021**, *49*, D1541–D1547. https://doi.org/10.1093/nar/gkaa1011

16. de Leeuw, C.A.; Mooij, J.M.; Heskes, T.; Posthuma, D. MAGMA: generalized gene-set analysis of GWAS data. *PLoS Comput. Biol.* **2015**, *11*, e1004219. https://doi.org/10.1371/journal.pcbi.1004219

17. Kerimov, N.; Hayhurst, J.D.; Peikova, K.; Manning, J.R.; Walter, P.; Kolberg, L.; Samoviča, M.; Sakthivel, M.P.; Kuzmin, I.; Trevanion, S.J.; Burdett, T.; Jupp, S.; Parkinson, H.; Papatheodorou, I.; Yates, A.D.; Zerbino, D.R.; Alasoo, K. A compendium of uniformly processed human gene expression and splicing quantitative trait loci. *Nat. Genet.* **2021**, *53*, 1290–1299. https://doi.org/10.1038/s41588-021-00924-w

18. Giambartolomei, C.; Vukcevic, D.; Schadt, E.E.; Franke, L.; Hingorani, A.D.; Wallace, C.; Plagnol, V. Bayesian test for colocalisation between pairs of genetic association studies using summary statistics. *PLoS Genet.* **2014**, *10*, e1004383. https://doi.org/10.1371/journal.pgen.1004383

19. Szklarczyk, D.; Kirsch, R.; Koutrouli, M.; Nastou, K.; Mehryary, F.; Hachilif, R.; Gable, A.L.; Fang, T.; Doncheva, N.T.; Pyysalo, S.; Bork, P.; Jensen, L.J.; von Mering, C. The STRING database in 2023: protein-protein association networks and functional enrichment analyses for any sequenced genome of interest. *Nucleic Acids Res.* **2023**, *51*, D638–D646. https://doi.org/10.1093/nar/gkac1000

20. Köhler, S.; Bauer, S.; Horn, D.; Robinson, P.N. Walking the interactome for prioritization of candidate disease genes. *Am. J. Hum. Genet.* **2008**, *82*, 949–958. https://doi.org/10.1016/j.ajhg.2008.02.013

21. Pedersen, H.K.; Gudmundsdottir, V.; Nielsen, H.B.; Hyotylainen, T.; Nielsen, T.; Jensen, B.A.; Forslund, K.; Hildebrand, F.; Prifti, E.; Falony, G.; Le Chatelier, E.; Levenez, F.; Doré, J.; Mattila, I.; Plichta, D.R.; Pöhö, P.; Hellgren, L.I.; Arumugam, M.; Sunagawa, S.; Vieira-Silva, S.; Jørgensen, T.; Holm, J.B.; Trošt, K.; MetaHIT Consortium; Kristiansen, K.; Brix, S.; Raes, J.; Wang, J.; Hansen, T.; Bork, P.; Brunak, S.; Oresic, M.; Ehrlich, S.D.; Pedersen, O. Human gut microbes impact host serum metabolome and insulin sensitivity. *Nature* **2016**, *535*, 376–381. https://doi.org/10.1038/nature18646

22. Shats, I.; Williams, J.G.; Liu, J.; Makarov, M.V.; Wu, X.; Lih, F.B.; Deterding, L.J.; Lim, C.; Xu, X.; Randall, T.A.; Lee, E.; Li, W.; Fan, W.; Li, J.L.; Sokolsky, M.; Kabanov, A.V.; Li, L.; Migaud, M.E.; Locasale, J.W.; Li, X. Bacteria Boost Mammalian Host NAD Metabolism by Engaging the Deamidated Biosynthesis Pathway. *Cell Metab.* **2020**, *31*, 564–579.e7. https://doi.org/10.1016/j.cmet.2020.02.001
